# Supplementary material for: The co-management of HIV and chronic non-communicable diseases in the Dominican Republic: A qualitative study
Source: PLoS One. 2023 Jul 13;18(7):e0288583. doi: 10.1371/journal.pone.0288583 (PMC10343047; doi:10.1371/journal.pone.0288583)
Supplement: S1 File — (PDF) [file pone.0288583.s001.pdf]

ProMeSA

End Line Semi-structured Interview Guide

Introduction: Thank you for meeting with me today. Today we are speaking with people who have received medical care at this clinic who are managing HIV and other chronic conditions such as elevated blood sugar levels. We are also interested in understanding more about your experiences participating in the ProMeSA program, specifically with the urban gardens and nutritional education. Please do not worry about remembering all the details. What I am interested in are your experiences and reactions in general about the topics we will talk about today and there are no correct answers.

As I explained in the consent, I will ask you questions and some may be sensitive. If you feel uncomfortable with any question, we can skip it. All the information you share with me today is confidential, meaning that only members of the study team will have access to it. I would like to audio record this interview to be able to later transcribe the interview and ensure that we accurately capture your comments. Once we complete the transcription, we will destroy the audio recording (approximately January or February 2020). I would like to emphasize that nothing that we will talk about will affect your HIV care. I will be taking notes during the interview so that I can better remember what we spoke about and to help with interpretation. I will not include your name in these notes to keep it confidential. Do you have any general questions for me before we start?

(PAUSE, ALLOW TIME FOR QUESTIONS)
I will **start the recorder** now.

Participant Identification Number: ________________________

SECTION 1: CONTEXT	
These questions will help me better understand you as a person.	
1.1	To start, I would like to learn more about you. Could you tell me about a typical day in your life – in terms of you what you eat, what are your primary activities, etc.? What do you do the moment you wake up in the morning until you go to bed at night? *[If necessary, you can separate what is done on a weekday compared to a weekend]*<br>PROBES:<br>• What do you eat for breakfast? Lunch? Dinner?<br>• What are some activities that you participate in typically?

<table>
<tr><th colspan="2">SECTION 2. HEALTH EXPERIENCES</th></tr>
<tr><td colspan="2">Thank you to speaking with me about your experiences managing your physical and mental health.</td></tr>
<tr><td>2.1</td><td>Before enrolling in the ProMeSA program, how was your physical health?
<u>PROBES:</u>
How did you think your overall physical health was (before enrolling in the program)?
Did you have a health condition, other than HIV, that you had to manage while you were participating in the program?
Elevated blood glucose? [Type 2 diabetes or prediabetes]
Overweight?
High blood pressure?
High cholesterol?
Others?</td></tr>
<tr><td>2.2</td><td>Before enrolling in the ProMeSA program, how would you describe your mental health?
<u>PROBES:</u>
How did you think your mental health was (before enrolling in the program)?
Did you have a mental health condition that you had to manage during your participation in the program?
Depression?
Anxiety?
Stress?
Other?</td></tr>
<tr><td>2.3</td><td>Reflecting on your experiences living with HIV, tell me about how you learned that you had HIV?
<u>PROBES:</u>
How did you learn that you had HIV? (where, when, under what circumstances)?
How did you feel when you were diagnosed?
What did you know about HIV at that time?
Did you know other people who had HIV at that time?
Who provided support when you were newly diagnosed with HIV?</td></tr>
<tr><td>2.4</td><td>At this time, how are you managing your HIV?
<u>PROBES:</u>
What is the easiest aspect of living with HIV?
What is the hardest aspect of living with HIV?
Who supports you in managing your HIV?</td></tr>
<tr><td>2.5</td><td>Reflecting on your experience with X [from 2.1: elevated blood sugar, diabetes/prediabetes, overweight, high blood pressure, high cholesterol or other], tell me more about how you learned that you had X?
<u>PROBES:</u>
How did you learn that you had X [elevated blood sugar, diabetes/prediabetes, overweight, high blood pressure, high cholesterol or other]? (where, when, under what circumstances)?
How did you feel when they told you?
What did you know about X [diabetes/prediabetes, overweight, high blood pressure, high cholesterol or other] at that time?
Did you know other people who had X [diabetes/prediabetes, overweight, high blood pressure, high cholesterol or other] at that time?
Who supported you when you were newly diagnosed with X [diabetes/prediabetes, overweight, high blood pressure, high cholesterol or other]?</td></tr>
<tr><td>2.6</td><td>Presently, how are you managing your X [diabetes/prediabetes, overweight, high blood pressure, high cholesterol or other]?
<u>PROBES:</u>
What is the easiest aspect of managing your X [diabetes/prediabetes, overweight, high blood pressure, high cholesterol or other]?</td></tr>
</table>

	• What is the hardest aspect of managing your X [diabetes/prediabetes, overweight, high blood pressure, high cholesterol or other]?<br>• Who is supporting you as you manage your X [diabetes/prediabetes, overweight, high blood pressure, high cholesterol or other]?
2.7	Which of your health conditions worries you the most? Why? *[This can include any health condition mentioned previously or others such as pain, an injury, etc. It does not need to be HIV or diabetes/elevated blood sugar]*
2.8	At this time, do you believe you are managing one condition (HIV or X) better than the other?<br>PROBES:<br>• Why do you think you can manage one condition better than the other?<br>• What do you need to better manage the condition you are most concerned about?<br>o Social support?<br>o Access to health services?<br>o Access to medications?

**SECTION 3. HEALTH SERVICES**	
Thank you for telling me about your health experiences. Now I am interested in understanding more about your experiences with the health care system.	
3.1	Could you describe what happens during a typical medical visit with your doctor?<br>PROBES:<br>• Do you normally see one doctor or do you see multiple doctors?<br>o If you have multiple doctors, who is your primary doctor?<br>• How does your doctor explain how to manage multiple health conditions?
3.2	Based on your experiences, do you believe it is easier to access health services for HIV or for X [diabetes/prediabetes, overweight, high blood pressure, high cholesterol or other]?
3.3	Tell me more about how it is to access mental health services.<br>• Where can you go for mental health services?<br>• Is it easy to find mental health providers?<br>• What are the barriers for finding mental health services?

SECTION 4. FEASIBILITY AND ACCEPTABILITY OF THE INTERVENTION	
Next, I would like to ask you about your opinions of the ProMeSA program.	
4.1	Tell me more about your general impressions with the ProMeSA program (urban gardens and nutritional counseling).<br>• What did you like the most about the program?<br>• What did you like the least about the program?<br>• What would you change about the program so that it could be better?
4.2	Tell me about the experiences you had with the urban gardens in the program [Clarify if the participant had a home garden, participated in a community garden, or did both or neither].<br><u>PROBES:</u><br>• Which aspects of the garden program did you like?<br>• Which aspects of the garden program did you not like?
4.3	What were the barriers you experienced in starting and maintaining your garden?<br>• How useful were the gardening classes in helping you start a garden?<br>• How useful was the follow-up by the agronomist to your home in establishing and maintaining your garden?<br>• What problems did you experience in having a garden (space, water, animals, diseases)? How were you able to resolve it, or not?<br>• What were the facilitators in having a garden? What helped you?
4.5	Tell me about the experiences you had with the nutritional counseling in the program (nutritional counseling from Romelia and the cooking classes).<br><u>PROBES:</u><br>• What aspects of participating in nutritional counseling did you like?<br>• What aspects of participating in nutritional counseling did you not like?
5.6	What were the barriers you faced in participating in nutritional education?<br>• What were the barriers for participating in the nutritional counseling in the SAI?<br>• What were the barriers for participating in the cooking workshops?<br>• How easy was it to apply your knowledge about nutrition and healthy cooking in your daily life?<br>• What were the facilitators in helping you participate in the nutritional education and apply the lessons to your own life?
4.7	How easy or difficult was it to participate in the data collection process for ProMeSA? In other words, the survey, weight measures, height measures, blood draws, etc.?<br>• What changes do you suggest that we make to these measurements?<br>• Was it difficult to come back to the clinic to get a blood draw?
4.8	Do you have other comments to improve the program for future participants?

SECTION 4. PERCEPTIONS OF CHANGES	
Next, I wanted to ask you about your thoughts on the ProMeSA program and your perceptions regarding what you have seen or experienced in the last 12 months as a participant in the program.	
5.1	Tell me about the foods you typically ate before you participated in the ProMeSA program.<br>PROBES:<br>• Describe your diet or what you normally ate before enrolling in the program?<br>• Reflecting on your experience in the program, what do you typically eat now?<br>• How did the types of foods you ate change as compared to before starting the program?<br>o Fruits??<br>o Vegetables?<br>o Grains?<br>What changed in terms of the quantity of foods as compared to before starting the program?
5.2	Before enrolling in the program, did you have any problems getting the amount of food you wanted or needed for you and your family?<br>PROBES:<br>• Did you have problems getting the variety of foods that you needed or wanted?
5.3	Could you tell me about the skills and knowledge you gained during the program?<br>PROBES:<br>• In relation to having a garden?<br>• In relation to healthy eating?<br>• In relation to managing your HIV and/or other conditions?
5.4	In thinking about the last 12 months since you started participating in the program:<br><br>Have you had any changes in your **quality of life**?<br>PROBES: *[If they said there were changes]*<br>• What do you attribute these changes in your quality of life to?<br>• What aspects of the program contributed to these changes?<br><br>Have you had any changes in your **social interactions** in your community?<br>PROBES: *[If they said there were changes]*<br>• What do you attribute these changes in your **social interactions** to?<br>• What aspects of the program contributed to these changes?<br><br>Have you experienced changes in your **relationships with your family**?<br>PROBES: *[If they said there were changes]*<br>• What do you attribute these changes in your **relationships with your family** to?<br>• What aspects of the program contributed to these changes?<br><br>Have you experienced changes in your relationship with your **medical provider**?<br>PROBES: *[If they said there were changes]*<br>• What do you attribute these changes in your relationship with your **medical provider** to?<br>• What aspects of the program contributed to these changes?<br><br>Have you had changes in your **physical health**?<br>PROBES: *[If they said there were changes]*<br>• What do you attribute these changes in your **physical health** to?<br>• What aspects of the program contributed to these changes?<br><br>Have had changes in your **mental health**?<br>PROBES: *[If they said there were changes]*

	• What do you attribute these changes in your **mental health** to?<br>• What aspects of the program contributed to these changes?<br><br>Have you had changes in your level of **finances**?<br>PROBES: *[If they said there were changes]*<br>• What do you attribute these changes in your level of **finances** to?<br>• What aspects of the program contributed to these changes?<br><br>Have you experienced changes in your **feelings about yourself**?<br>PROBES: *[If they said there were changes]*<br>• What do you attribute these changes in your **feelings about yourself** to?<br>• What aspects of the program contributed to these changes?

**SECTION 6. CONCLUSION**	
Thank you again for your participation, I have a couple more questions and then we are done.	
6.1	We are reaching the end of the interview. Are there any additional details you would like to add to any of the topics we discussed today?
6.2	Do you have any other questions for me before we finish the interview?
