# Supplementary material for: The co-management of HIV and chronic non-communicable diseases in the Dominican Republic: A qualitative study
Source: PLoS One. 2023 Jul 13;18(7):e0288583. doi: 10.1371/journal.pone.0288583 (PMC10343047; doi:10.1371/journal.pone.0288583)
Supplement: Attachment — Submitted filename: PONE-D-22-12189_R1_reviewer.pdf [file pone.0288583.s004.pdf]

PLOS ONE

The co-management of HIV and chronic non-communicable diseases in the Dominican Republic: A qualitative study

--Manuscript Draft--

Manuscript Number:	PONE-D-22-12189R1
Article Type:	Research Article
Full Title:	The co-management of HIV and chronic non-communicable diseases in the Dominican Republic: A qualitative study
Short Title:	Disease co-management in the Dominican Republic
Corresponding Author:	Deshira Wallace, Ph.D.<br>University of North Carolina at Chapel Hill Gillings School of Global Public Health<br>Chapel Hill, NC UNITED STATES
Keywords:	HIV care; chronic disease care; Diabetes; hypertension; narrative; Dominican Republic; urban
Abstract:	People living with HIV and a non-communicable disease (NCD) experience multi-level barriers when co-managing both conditions. We explored the factors affecting living with multiple chronic conditions in the Dominican Republic. We conducted 21 in-depth interviews from October 2019-February 2020 with Dominican adults experiencing food insecurity and managing HIV and at least one chronic NCD. Using thematic analysis, we explored participant lived experiences co-managing multiple chronic conditions. All participants (mean age = 45.5 years) were linked to HIV care, but only three were linked to NCD-specific care. Individual-level barriers to managing NCDs included limited education and limited self-efficacy for self-management. Interpersonally, barriers included limited rapport building with an NCD-specific specialist. Structurally, barriers to managing NCDs were no health insurance, poor referral systems, and limited financial assistance. Health system adaptation requires equitably considering the needs of individuals managing multiple chronic conditions. Key factors to address include patient-provider relationships, improved referral systems, accessibility and availability of specialists, and financial assistance.
Order of Authors:	Deshira D Wallace, Ph.D.
	Amarilis Then-Paulino, MD
	Gipsy Jiménez Paulino, MD
	Flabia Tejada Castro, MD
	Stephanie Daniela Castro, MD
	Kartika Palar, PhD
	Kathryn P Derose, PhD
Response to Reviewers:	Dear Editorial team,<br>We thank you for the opportunity to resubmit the manuscript: “The co-management of HIV and chronic non-communicable diseases in the Dominican Republic: A qualitative study” for exclusive consideration in PLOS One.<br>Our response to reviewers is found below.<br><br>Editorial Notes<br>When submitting your revision, we need you to address these additional requirements.<br>1.Please ensure that your manuscript meets PLOS ONE's style requirements, including those for file naming. The PLOS ONE style templates can be found at https://journals.plos.org/plosone/s/file?id=wjVg/PLOSOne_formatting_sample_main_body.pdf and<br>https://journals.plos.org/plosone/s/file?id=ba62/PLOSOne_formatting_sample_title_authors_affiliations.pdf<br>•We have ensured that we followed the PLOS ONE style requirements.<br>2.Please include a complete copy of PLOS’ questionnaire on inclusivity in global research in your revised manuscript. Our policy for research in this area aims to

Powered by Editorial Manager® and ProduXion Manager® from Aries Systems Corporation

improve transparency in the reporting of research performed outside of researchers' own country or community. The policy applies to researchers who have travelled to a different country to conduct research, research with Indigenous populations or their lands, and research on cultural artefacts. The questionnaire can also be requested at the journal's discretion for any other submissions, even if these conditions are not met. Please find more information on the policy and a link to download a blank copy of the questionnaire here: https://journals.plos.org/plosone/s/best-practices-in-research-reporting. Please upload a completed version of your questionnaire as Supporting Information when you resubmit your manuscript.
•The questionnaire on inclusivity in global research was completed and uploaded.

3.Please provide additional details regarding participant consent. In the ethics statement in the Methods and online submission information, please ensure that you have specified whether: 1) whether the ethics committee approved the verbal/oral consent procedure, 2) why written consent could not be obtained, and 3) how verbal/oral consent was recorded. If your study included minors, please state whether you obtained consent from parents or guardians in these cases. If the need for consent was waived by the ethics committee, please include this information.
•Consent information was updated. We specifically added text on why we conducted verbal consent (for literacy and accessibility purposes).

4.In your Data Availability statement, you have not specified where the minimal data set underlying the results described in your manuscript can be found. PLOS defines a study's minimal data set as the underlying data used to reach the conclusions drawn in the manuscript and any additional data required to replicate the reported study findings in their entirety. All PLOS journals require that the minimal data set be made fully available. For more information about our data policy, please see http://journals.plos.org/plosone/s/data-availability.
"Upon re-submitting your revised manuscript, please upload your study's minimal underlying data set as either Supporting Information files or to a stable, public repository and include the relevant URLs, DOIs, or accession numbers within your revised cover letter. For a list of acceptable repositories, please see http://journals.plos.org/plosone/s/data-availability#loc-recommended-repositories. Any potentially identifying patient information must be fully anonymized.
Important: If there are ethical or legal restrictions to sharing your data publicly, please explain these restrictions in detail. Please see our guidelines for more information on what we consider unacceptable restrictions to publicly sharing data: http://journals.plos.org/plosone/s/data-availability#loc-unacceptable-data-access-restrictions. Note that it is not acceptable for the authors to be the sole named individuals responsible for ensuring data access.
We will update your Data Availability statement to reflect the information you provide in your cover letter.
•We have added a statement on data availability of the following: "Because we did not state in the consent form that the data could be made available to outside researchers, our IRB has informed us that we cannot make the data available outside the study team."
•Therefore, we cannot provide a URL for public access to the data.

Based on the November 16th message from the PLOS One Editorial Team:
Comment
1. We note your updated Data Availability statement as follows:
"Data underlying the results cannot be shared publicly to protect participant privacy and confidentiality. Because we did not state in the consent form that the data could be made available to outside researchers, our IRB has informed us that we cannot make the data available outside the study team."

In this instance it seems there may be acceptable restrictions in place that prevent the public sharing of your minimal data, however, in line with our goal of ensuring long-term data availability to all interested researchers, PLOS' Data Policy states that authors cannot be the sole named individuals responsible for ensuring data access (http://journals.plos.org/plosone/s/data-availability#loc-acceptable-data-sharing-methods).

Data requests to a non-author institutional point of contact, such as a data access or

ethics committee, helps guarantee long term stability and availability of data. Providing interested researchers with a durable point of contact ensures data will be accessible even if an author changes email addresses, institutions, or becomes unavailable to answer requests.

Before we proceed with your manuscript, please address the following prompts:

a) If there are ethical or legal restrictions on sharing a de-identified data set, please explain the restrictions in detail (e.g., data contain potentially identifying or sensitive patient information) and who has imposed them (e.g., a Research Ethics Committee or Institutional Review Board, etc.). Please also provide non-author contact information (phone/email/hyperlink) for a data access committee, ethics committee, or other institutional body to which data requests may be sent. If applicable, please also provide any necessary information which interested researchers would need when requesting access to data in order to obtain the minimal data set for your study.

We will update your Data Availability statement on your behalf to reflect the information you provide.

Response
"The data upon which this manuscript is based are in-depth interview transcripts from a small sample of marginalized and stigmatized people with HIV and contain highly sensitive data that are identifiable via inference. Thus, sharing them would violate the promise of confidentiality made to participants during informed consent. Because we did not state in the consent form that the data could be made available to outside researchers, the RAND Human Subjects Protection Committee (HSPC) has informed us that we cannot make the data available outside the study team. Queries regarding data access can be addressed by the RAND HSPC by contacting Rebecca Collins, HSPC Chair (collins@rand.org)."

Comments to the Author

Reviewer #1:
The co-management of HIV and chronic non-communicable diseases in the Dominican
Republic: A qualitative study

Review:

Manuscript presented an important topic and problem of high significance. The authors intended to explore PLWH lived experiences in co-managing multiple chronic conditions. There are significant and minor revisions suggested and described in each section.

Background
There is the mention of food insecurity in the background but not much is discussed about it. Is this a problem in Dominican Republic? Who is severely affected by food insecurity? Context can help guide the reason for including food insecurity.
•Thank you for this critique. We have added additional context and information about food security in lines 74-81. We included additional citations that focus on the role food insecurity has on the management of different conditions (HIV, NCDs).

Line 25-27 - “Of the individuals living with HIV in the Dominican Republic, 82% know their status and 51% of people living HIV, regardless of status, are on antiretroviral therapy (ART)”
This sentence is not clear. “Regardless of status” Does this mean that there are some without HIV and are accounted for in the 51%? Please assist the reader to understand the reason why it is important to include those without HIV and ART.
•The line in question was edited for clarity.

Line 31-38 described overall trends of NCDs with little mention of the current situation in Dominican Republic. For example, are the age groups of those with HIV changing to

Powered by Editorial Manager® and ProduXion Manager® from Aries Systems Corporation

	an older population? This can help motivate the significance of the research area.<br>•Lines 34-37 are specifically referring to the overall trends in the Dominican Republic and lines 21-29 refers to the current situation in the Dominican Republic in regard to HIV.<br><br>Line 45-46: "At the individual level, those with multiple conditions require greater healthcare utilization and therefore incur greater healthcare costs than individuals with one or zero chronic conditions."<br>Is this to be required or studies have found association between number of chronic conditions and higher utilization and cost?<br>•We have added a citation to validate the argument.<br><br>Lines 39-69: Appreciated the background information but this section was hard to follow and also was too general. It would be helpful to narrow the focus to HIV and NCD. Discussing the conditions separately was very hard to tie back to the objective of the study. Please review this section.<br>•Thank you for your comments. We reviewed this section and note that it is focused on the discussion of HIV and NCDs, specifically the need to consider the co-management of these conditions. Lines 17-37 do present the context of these conditions separately; however, the section indicates that the critique is focused on the interplay of these conditions. Therefore, we did not make any edits beyond those for clarity and readability.<br><br>Line 79 mentions the use of the socio ecological model to inform the study. In the background section, one expects to learn how the model has been used in other studies and then its choice is defended in the methods section. Was the model used to develop the interview guide? Why was this the model of choice?<br>•The socioecological model is a framing for the data collection strategy and analysis. It's been used extensively in public health, but to clarify for the reader on how we used it, we added a sentence to the Data Collection subsection that reads as follows: "Using a socioecological model we framed question to understand individual, interpersonal, organizational, and political barriers and facilitators to managing multiple chronic conditions." Also, in lines 66-68 we reference the need to account for "individual, interpersonal, and structural facilitators and barriers to disease co-management…" which alludes to the socioecological model.<br><br>Methods<br>Line 107- there is a mention of a "ProMeSA study" this is the first time it is mentioned and the last time. What was it? How was it related to the intervention?<br>•The ProMeSA study is the intervention study. We added language to clarify that the intervention study is named ProMeSA. Related information about the study was masked for review, however, we have cited the intervention for more information.<br>•The endline interview are the data used for the present substudy, as explained in lines 109-117.<br><br>Why did the researchers decide to interview participants in the intervention group? Since they were interested in barriers and facilitators for co-management of NCDs, what was specific about the intervention group?<br>•All participants were part of an intervention, thus there is not a differentiation between a non-intervention and intervention group. The intervention, ProMeSA, components was described in lines 95-101. We chose to do a qualitative evaluation of the intervention overall, but also asked about barriers and facilitators for co-managing multiple chronic conditions. Therefore, as the main study only targeted HIV-specific outcomes, we could ask about NCD self-management in the qualitative sub-study<br><br>Recommend use the COREQ (COnsolidated criteria for REporting Qualitative research) Checklist as a guide in what to be reported.<br>•Thank you for the recommendation, we have filled out the COREQ checklist.<br><br>Results<br>Line 117: mentions of recruitment until saturation was reached. After how many participants was saturation reached?<br>•At approximately 20 participants we did reach thematic saturation, therefore we stopped recruitment at 21 participants.

Powered by Editorial Manager® and ProduXion Manager® from Aries Systems Corporation

Generally: although food insecurity is mentioned in the background, nothing seemed to have been explored in the topic.
•Thank you for this comment. The intervention study enrolled only participants who had moderate or severe food insecurity (per the Latin American Food Insecurity Scale). As this is the case, all narratives (i.e., data) are from this lens. We have added text in the results to this effect (lines 179-181). We also ensured to emphasize this throughout the manuscript to strengthen this foundation.

Since the study was focused on co-management of HIV and chronic NCDs, it is important that the results mostly focus on this aspect. The sections beginning line 174 - 239 seem to be out of the topic of discussion.
•Thank you for this comment. We understand that the focus is on the co-management of HIV and NCDs. We felt that it was important to provide some context for what it is like to live with HIV for adults who are food insecure in general, and equally what it is like to live with NCDs as an adult experiencing food insecurity.

Minor: In table 2, authors should be consistent with abbreviations (T2D was used in some places and then one would see "type 2 diabetes" later)
•Thank you. We have corrected Table 2 for consistency.

Line 293 -294 "The lack of education was also tied to how participants experienced their condition as being "sensitive" and "unpredictable" from one day to the next."
This sentence is not clear. Consider revising.
•Thank you. We clarified what we meant by lack of education. The sentence now reads as follows: The lack of education on NCD self-management was also tied to how participants experienced their condition as being "sensitive" and "unpredictable" from one day to the next.

Line 327-337: this section was confusing. It is not clear what was said by participants.
•Thank you for your note. This first paragraph under structural level concerns focused on summarizing results from demographic variables (see Table 1). We also made a note throughout this paragraph that compared how participants spoke about their experiences with accessing the health care system. These experiences did not change much between those who were insured or not, particularly when managing NCDs. Therefore, we did not pull specific quotes. Rather we highlighted exemplar experiences for the remaining parts of the subsection.

Line 332-334 is also confusing. What information should the reader get from this sentence? "Of the five participants who were without health insurance …….three were the participants of Haitian descent"
Why was it important for the authors to add this sentence? Should the reader be introduced to the aspect earlier in the background?
•At the beginning of the results section and Table 1, we have an ancestry break down in which three participants from our total pool of participants were of Haitian descent. We brought up this sentence of lack of health insurance in the results and further link to potential implications of this in second paragraph of the discussion section. In the discussion we note potential barriers for Haitians in the Dominican Republic to gain access to health insurance. We did not bring this up in the background because this was not an a priori assumption and it fit best to link the results to the discussion.

Discussion
The discussion section provided information about that healthcare system that should have been provided in the background section. Consider revising this section to discuss the results and next steps.
•Thank you for this comment. The reason we have included the background information on the healthcare system in the discussion rather than in the background is because it was related to how participants described their experiences. This was not an a priori assumption, and we wanted to recognize this as linked to the data.

Reviewer #2:
1.Well done overall. The presentation and discussion on the key facilitators and barriers for individuals living with HIV and co-occurring NCDs was informative -

Powered by Editorial Manager® and ProduXion Manager® from Aries Systems Corporation

	especially Table 2.<br>•Thank you for the comments. We appreciate that the manuscript was considered relevant and informative.<br><br>2.Please re-read the manuscript as there are a few grammatical errors within the text.<br>•We have re-read the manuscript and addressed grammatical errors.
**Additional Information:**	
**Question**	**Response**
**Financial Disclosure**<br><br>Enter a financial disclosure statement that describes the sources of funding for the work included in this submission. Review the submission guidelines for detailed requirements. View published research articles from *PLOS ONE* for specific examples.<br><br>This statement is required for submission and **will appear in the published article** if the submission is accepted. Please make sure it is accurate.<br><br>**Unfunded studies**<br>Enter: *The author(s) received no specific funding for this work.*<br><br>**Funded studies**<br>Enter a statement with the following details:<br>• Initials of the authors who received each award<br>• Grant numbers awarded to each author<br>• The full name of each funder<br>• URL of each funder website<br>• Did the sponsors or funders play any role in the study design, data collection and analysis, decision to publish, or preparation of the manuscript?<br>• **NO** - Include this sentence at the end of your statement: *The funders had no role in study design, data collection and analysis, decision to publish, or preparation of the manuscript.*<br>• **YES** - Specify the role(s) played.<br><br>* typeset	Research reported in this publication was supported by the National Institute of Mental Health of the National Institutes of Health under Award Number R34MH110325 (KPD). DW was supported by NICHD of the National Institutes of Health under award number P2C HD050924. KP contributions were supported by a grant from the National Institutes of Health, University of California, San Francisco-Gladstone Institute of Virology & Immunology Center for AIDS Research, P30AI027763 (NIAID). The article contents are solely the responsibility of the authors and do not represent the official views of the National Institutes of Health. The funders had no role in study design, data collection and analysis, decision to publish, or preparation of the manuscript.
**Competing Interests**<br><br>Use the instructions below to enter a competing interest statement for this submission. On behalf of all authors, disclose any competing interests that	The authors have declared that no competing interests exist.

could be perceived to bias this work—acknowledging all financial support and any other relevant financial or non-financial competing interests.<br><br>This statement is required for submission and **will appear in the published article** if the submission is accepted. Please make sure it is accurate and that any funding sources listed in your Funding Information later in the submission form are also declared in your Financial Disclosure statement.<br><br>View published research articles from *PLOS ONE* for specific examples.<br><br>**NO authors have competing interests**<br><br>Enter: *The authors have declared that no competing interests exist.*<br><br>**Authors with competing interests**<br><br>Enter competing interest details beginning with this statement:<br><br>*I have read the journal's policy and the authors of this manuscript have the following competing interests: [insert competing interests here]*<br><br>* typeset	
**Ethics Statement**<br><br>Enter an ethics statement for this submission. This statement is required if the study involved:<br><br>• Human participants<br>• Human specimens or tissue<br>• Vertebrate animals or cephalopods<br>• Vertebrate embryos or tissues<br>• Field research<br><br>Write "N/A" if the submission does not require an ethics statement.	The RAND Corporation institutional review board and the Universidad Autonoma de Santo Domingo ethics board and the Dominican Ministry of Health approved this study. Participants provided oral consent for the study.

Powered by Editorial Manager® and ProduXion Manager® from Aries Systems Corporation

General guidance is provided below. Consult the submission guidelines for detailed instructions. **Make sure that all information entered here is included in the Methods section of the manuscript.**<br><br>**Format for specific study types**<br><br>**Human Subject Research (involving human participants and/or tissue)**<br>• Give the name of the institutional review board or ethics committee that approved the study<br>• Include the approval number and/or a statement indicating approval of this research<br>• Indicate the form of consent obtained (written/oral) or the reason that consent was not obtained (e.g. the data were analyzed anonymously)<br><br>**Animal Research (involving vertebrate animals, embryos or tissues)**<br>• Provide the name of the Institutional Animal Care and Use Committee (IACUC) or other relevant ethics board that reviewed the study protocol, and indicate whether they approved this research or granted a formal waiver of ethical approval<br>• Include an approval number if one was obtained<br>• If the study involved *non-human primates*, add *additional details* about animal welfare and steps taken to ameliorate suffering<br>• If anesthesia, euthanasia, or any kind of animal sacrifice is part of the study, include briefly which substances and/or methods were applied<br><br>**Field Research**<br><br>Include the following details if this study involves the collection of plant, animal, or other materials from a natural setting:<br>• Field permit number<br>• Name of the institution or relevant body that granted permission	
**Data Availability**<br><br>Authors are required to make all data	No - some restrictions will apply

Powered by Editorial Manager® and ProduXion Manager® from Aries Systems Corporation

underlying the findings described fully available, without restriction, and from the time of publication. PLOS allows rare exceptions to address legal and ethical concerns. See the PLOS Data Policy and FAQ for detailed information.<br><br>A Data Availability Statement describing where the data can be found is required at submission. Your answers to this question constitute the Data Availability Statement and **will be published in the article**, if accepted.<br><br>**Important:** Stating 'data available on request from the author' is not sufficient. If your data are only available upon request, select 'No' for the first question and explain your exceptional situation in the text box.<br><br>Do the authors confirm that all data underlying the findings described in their manuscript are fully available without restriction?	
**Describe where the data may be found in full sentences. If you are copying our sample text, replace any instances of XXX with the appropriate details.**<br><br>• If the data are **held or will be held in a public repository**, include URLs, accession numbers or DOIs. If this information will only be available after acceptance, indicate this by ticking the box below. For example: *All XXX files are available from the XXX database (accession number(s) XXX, XXX.).*<br>• If the data are all contained **within the manuscript and/or Supporting Information files**, enter the following: *All relevant data are within the manuscript and its Supporting Information files.*<br>• If neither of these applies but you are able to provide **details of access elsewhere**, with or without limitations, please do so. For example:<br><br>*Data cannot be shared publicly because of [XXX]. Data are available from the*	The data upon which this manuscript is based are in-depth interview transcripts from a small sample of marginalized and stigmatized people with HIV and contain highly sensitive data that are identifiable via inference. Thus, sharing them would violate the promise of confidentiality made to participants during informed consent. Because we did not state in the consent form that the data could be made available to outside researchers, the RAND Human Subjects Protection Committee (HSPC) has informed us that we cannot make the data available outside the study team. Queries regarding data access can be addressed by the RAND HSPC by contacting Rebecca Collins, HSPC Chair (collins@rand.org).

XXX Institutional Data Access / Ethics Committee (contact via XXX) for researchers who meet the criteria for access to confidential data.<br><br>*The data underlying the results presented in the study are available from (include the name of the third party and contact information or URL).*<br>• This text is appropriate if the data are owned by a third party and authors do not have permission to share the data.<br><br>* typeset	
Additional data availability information:	

Powered by Editorial Manager® and ProduXion Manager® from Aries Systems Corporation

The co-management of HIV and chronic non-communicable diseases in the Dominican Republic: A qualitative study

Deshira D. Wallace[1*¶], Amarilis Then-Paulino[2&], Gipsy Jiménez Paulino[3&], Flabia Tejada Castro[4&], Stephanie Daniela Castro[5&], Kartika Palar[6&], Kathryn P. Derose[7,8¶]

[1]Department of Health Behavior, Gillings School of Global Public Health, University of North Carolina at Chapel Hill, Chapel Hill, North Carolina, United States of America

[2]Escuela de Salud Pública, Facultad de Ciencias de la Salud, Universidad Autónoma de Santo Domingo, Alma Máter, Ciudad Universitaria, Santo Domingo, Dominican Republic

[3]Viceministerio de Garantía de la Calidad, Ministerio de Salud Pública, Santo Domingo, Dominican Republic

[4]Independent Consultant, Santo Domingo, Dominican Republic

[5]Center for Diagnosis, Advanced Medicine, and Telemedicine (CEDIMAT), Santo Domingo, Dominican Republic

[6]Department of Medicine, Division of HIV, Infectious Disease, and Global Medicine, University of California, San Francisco, San Francisco, California, United States of America

[7]Department of Health Promotion & Policy, University of Massachusetts Amherst, Amherst, Massachusetts, United States of America

[8]Department of Behavioral and Policy Sciences, RAND Corporation, Santa Monica, California, United States of America

*Corresponding author

Email: ddwallac@email.unc.edu (DDW)

[¶]These authors contributed equally to this work.

[&]These authors also contributed equally to this work.

Abstract

People living with HIV and a non-communicable disease (NCD) experience multi-level barriers when co-managing both conditions. We explored the factors affecting living with multiple chronic conditions in the Dominican Republic. We conducted 21 in-depth interviews from October 2019-February 2020 with Dominican adults experiencing food insecurity and managing HIV and at least one chronic NCD. Using thematic analysis, we explored participant lived experiences co-managing multiple chronic conditions. All participants (mean age = 45.5 years) were linked to HIV care, but only three were linked to NCD-specific care. Individual-level barriers to managing NCDs included limited education and limited self-efficacy for self-management. Interpersonally, barriers included limited rapport building with an NCD-specific specialist. Structurally, barriers to managing NCDs were no health insurance, poor referral systems, and limited financial assistance. Health system adaptation requires equitably considering the needs of individuals managing multiple chronic conditions. Key factors to address include patient-provider relationships, improved referral systems, accessibility and availability of specialists, and financial assistance.

Introduction

The Caribbean region has been undergoing a health transition in which traditional infectious diseases have been occurring alongside with a rise of chronic non-communicable diseases (NCDs). As of 2019 approximately 1.1% of people in the Caribbean are living with HIV, making it the region with the second highest HIV prevalence after sub-Saharan Africa [1, 2]. Within the Caribbean, the Dominican Republic has a similarly high HIV prevalence comparable to the region at approximately 0.9% of the population [3, 4]. In fact, HIV/AIDS-related complications are the fifth cause of death in the country [5]. Of the individuals living with HIV in the Dominican Republic, 82% know their status; further, 51% of people living HIV, are on antiretroviral therapy (ART) [3]. Although HIV is an infectious disease, advances in treatment have shifted HIV to function more as a chronic condition, requiring similar behaviors for its management such as medication adherence, lifestyle adjustments, consistent and long-term provider interaction, and adequate interpersonal support [6].

As related to chronic NCDs, diabetes mellitus and cardiovascular diseases are among the leading causes of death and morbidity globally [7]. Chronic NCDs include a range of conditions such as cardiovascular diseases, type 2 diabetes mellitus (T2D), cancer, pulmonary diseases, neurological conditions, and mental health conditions. Trends in the global rise of chronic NCDs are similar in the Dominican Republic [8]. Cardiovascular diseases (ischemic heart disease, intracerebral hemorrhage, ischemic stroke) are three of the four top causes of death in the Dominican Republic [5]. Overall chronic NCDs accounted for 72% of deaths in the Dominican Republic in 2019, a 40% increase since 2010 [9].

With HIV being managed as a chronic condition, and the continued rise of chronic NCDs, there is acknowledgement that these conditions do not happen in silos. Thus, a shift in models of care that account for the management of multiple chronic conditions is necessary. However, as individuals with multiple chronic conditions represent an increasing proportion of

the adult population worldwide, studies have found that multilevel barriers affect the effectiveness of care for these individuals [10, 11]. At the individual level, those with multiple chronic conditions require greater healthcare utilization and therefore incur greater healthcare costs than individuals with one or zero chronic conditions [12]. The costs associated with high utilization can stem from emergency room use, inpatient hospitalizations, outpatient consultations and care, and medication use [10]. The cost of managing HIV and chronic NCDs disproportionately affect individuals who are socially and economically marginalized. Specifically, self-management for persons experiencing intersecting marginalized social and economic conditions, such as food insecurity, require using a significantly higher proportion of their available income compared to individuals who are economically secure and do not experience various social forms of marginalization and stigma [13, 14]. Interpersonally, individuals with multiple chronic conditions require sufficient social support from family, friends, and their healthcare team (i.e., patient-provider relationships) to manage their care [15, 16]. At the structural level, the lack of coordination between primary care providers and specialists, poor coordination among specialists to ensure suggested treatments are aligned, and healthcare accessibility (both distance and financial), and availability are important for the management of each condition [10]. These barriers are further complicated in low-and-middle income countries (LMICs) where the health systems are focused on addressing burdens of communicable diseases such as vector-borne conditions and HIV, as well as maternal, child and neonatal health [17]. Studies from Ethiopia and the United States have found an increasing number of people living with HIV (PLHIV) are not meeting glycemic control targets [18-20]. A study among 340 PLHIV in Brazil found a significant number of respondents were dealing with obesity, high cholesterol, and a significantly higher likelihood of metabolic syndrome [21]. A recent meta-analysis estimated that the regional prevalence of hypertension among PLHIV in Latin America and the Caribbean at 22% (17.8-26.5) indicating the increasingly overlapping health needs of PLHIV [22, 23]. Therefore, accounting for individual, interpersonal, and

structural facilitators and barriers to disease co-management is needed when evaluating living with multiple chronic conditions.

Although there are rich data on the experiences of managing HIV across several sub-populations in the Dominican Republic (e.g., urban versus rural, by occupation) [24-27], and there are increasing numbers of studies on T2D and cardiovascular diseases in the Dominican Republic [28-30]; to our knowledge, there is little data on the co-management of these conditions, particularly in a country with substantial investment in HIV care while balancing the rising burden of NCDs. Furthermore, food insecurity has been shown to affect management of both HIV and chronic NCDs such as T2D. In fact, food insecurity adversely affects HIV outcomes and NCDs through a variety of behavioral, nutritional, and psychosocial pathways [31, 32]. Understanding the realities of adults in structurally vulnerable food insecurity states, can improve our understanding of ways to adapt systems to improve disease co-management for broader segments of the population. Therefore, we were interested in exploring how adults experiencing food insecurity, and thus living in more resource-constrained environments, are managing multiple conditions in the Dominican Republic.

We use the socioecological model to inform this study, particularly in the exploration of the multidimensional nature of facilitators and barriers to living with and managing multiple chronic conditions in urbanized areas in the Dominican Republic. Specifically, we conducted qualitative interviews to explore the lived experiences of adults in the Dominican Republic who are experiencing food insecurity and managing both HIV and at least one chronic NCD and constructed themes in relation to individual level, interpersonal level, and structural level factors affecting their health and wellbeing. The contribution of this work is that it provides formative information on the considerations of public health approaches to address multiple condition management in a Dominican or similar contexts.

Materials and methods

Study

This qualitative study is part of an endline evaluation of a 12-month pilot intervention led by researchers at the RAND Corporation, a research non-profit organization, and the School of Health Sciences at the Universidad Autónoma de Santo Domingo (UASD). The intervention (i.e., ProMeSA) integrated urban gardens and peer nutritional counseling for adults with HIV experiencing moderate or severe food insecurity and treatment adherence difficulties in urban centers in the Dominican Republic [33]. The intervention comprised gardening training, home-based agricultural follow-up, peer nutritional counseling, and a group garden-based cooking workshop. The participants underwent two follow-up periods to assess potential changes HIV clinical outcomes (viral load, ART adherence, and HIV care retention) and other HIV-related outcomes (internalized and experienced stigma, social support).

The endline qualitative study involved in-depth interviews with patients participating in the pilot program at the intervention clinic (another clinic served as a usual care control). The broader goal of the interviews involved exploring health care experiences, facilitators and barriers to managing HIV, facilitators and barriers to managing a chronic NCD, and participants' perceptions regarding the feasibility, acceptability, and impacts of the intervention. This article focuses on the facilitators and barriers of co-managing HIV and a chronic NCD.

Setting and participants

The intervention study, ProMeSA, was conducted at two HIV clinics located in the central and northwestern part of the Dominican Republic [33]. The clinics are approximately a 2-hour drive from each other to avoid cross-contamination between participants. Both clinics were

selected as they were of similar sizes 500-800 patients on ART and offered similar standards of care. Participants in the intervention study were 18 years and older, registered patient at the HIV clinic sites, resided in an urban or peri-urban area, had moderate or severe food insecurity, and had evidence of adherence difficulties. For the qualitative endline sub-study, we used in-person purposive sampling [34] to select intervention participants who also had a chronic NCD (i.e., hypertension, diabetes, prediabetes, high cholesterol, obesity) according to previously collected data. We purposively recruited approximately equal numbers of men and women. Furthermore, participants were recruited until we reached thematic saturation. Institutional Review Boards and ethics committees from RAND, UASD, and the Dominican Ministry of Public Health approved the study protocols. All intervention study participants provided written consent; for the additional qualitative endline interview, participants provided verbal consent to account for literacy and accessibility.

Data collection

Three Dominican medical school graduates (one as intervention study coordinator [GJP] and two as research assistants [FTC, SDC]) were trained in qualitative data collection and analysis by KD and DW prior to and throughout the study period. This involved virtual and in-person trainings in Santo Domingo on how to conduct semi-structured interviews, manage data, transcribe audio recordings, and code transcripts. We used semi-structured, in-depth interviews to explore our themes of interest for this study: health experiences living with HIV, health experiences living with a chronic NCD, and health services experiences with both conditions. Using a socioecological model, we framed questions to understand individual, interpersonal, organizational, and political barriers and facilitators to managing multiple chronic conditions. Example questions included: how are you managing your HIV? How are you managing your chronic condition (T2D, high blood pressure)? Which of your health conditions worries you the most?

Qualitative interviews were completed between October 2019 – February 2020 at the intervention clinic in the Dominican Republic. All interviews were conducted in Spanish, lasted between 45-90 minutes, and were audio recorded with participant consent. After each interview, interviewers wrote field notes about the interview setting, key themes of the interview, observations for the following interviews, and if any themes may have reached saturation. Audio recordings were transcribed verbatim by the three trained interviewers/research assistants and verified by the first author.

Data analysis

We used a thematic analysis approach to explore participants' lived experiences co-managing multiple health conditions through the examination of deductive and inductive codes [35]. To do that, we first uploaded transcripts to Dedoose, a web-based text management and analysis software [36]. We then developed a codebook, which was initially developed using questions from the interview guide and field note observations. The codebook was iteratively updated based on the coding of initial transcripts. Four trained coders (DW, GJP, FTC, DC) read and coded the transcripts independently and the first author conducted a secondary coding review. Transcripts coded by the first author were reviewed by another member of the coding team. When reading the transcripts, coders wrote memos, or notes, about their observations of the data and linked what they read with other transcripts. All coding and analyses were completed in Spanish. Any discrepancies or questions about coding were resolved via coder consensus.

Some key codes we used to analyze participant experiences were: how participants were diagnosed with HIV and the chronic NCD, emotional reactions to their diagnoses, their understanding/knowledge of either condition, how they managed their conditions, social support with management, and their experiences with the healthcare system for both conditions. After

coding, all codes were placed in a matrix organized by participant to facilitate constant comparison of experiences among participants and within codes. We constructed summaries by code to examine similarities and differences in participant experiences, and then combined codes to construct overarching themes. Themes are organized using a socioecological framework approach, with consideration for individual, interpersonal, and structural facilitators and barriers to disease co-management in the Dominican Republic.

Results

A total of 21 participants were recruited, of which 12 were men and 9 were women. The mean age of participants was 45.5 years old. Eighteen participants self-identified as being of Dominican descent and three self-identified as being of Haitian descent. While all participants were diagnosed with HIV, participants had a range of chronic NCDs. These conditions included T2D, hypertension, high cholesterol, and cardiovascular and circulatory diseases (see Table 1). Note that individuals could be listed for multiple chronic NCDs.

Table 1. Participant demographics (n=21)

Variable	N	%	Mean
Age (years)			45.5
Female	9	42.9	
Ancestry			
Dominican	18	85.7	
Haitian	3	14.3	
Active Insurance Coverage	14	66.7	
Non-Communicable Chronic Conditions			
Prediabetes	5	23.8	
Diabetes	5	23.8	
Overweight/Obesity	12	57.1	
Hypertension	7	33.3	
High Cholesterol	3	14.3	
Cardiovascular disease	1	4.8	
Thrombosis	1	4.8	
Number of Chronic Conditions			

1	10	47.6	
2	7	33.3	
3	4	19.0	

The results are organized by providing a narrative of HIV management, narrative of chronic NCD management, and how these conditions compare in terms of their ability to co-manage them at the individual, interpersonal, and structural levels. Since only individuals who had experienced moderate or severe food insecurity were enrolled in the intervention study, all narratives are filtered through those experiences.

Living with HIV

Diagnosis. Participant narratives all began at diagnosis. All but three participants were diagnosed at a clinic or hospital, and the remaining three had a workplace screening that triggered a clinic referral. The reasons for initial HIV testing varied by gender with women describing getting tested for two reasons. The first was getting tested because their partner or spouse received a positive test, had HIV-related complications, or passed away due to untreated HIV complications. The second reason was pregnancy as two women tested positive after their second trimester tests. In contrast, men often got tested because they fell sick and went to a clinic where they learned their status and a few men went in for regular HIV/STI testing and received their diagnosis then.

Relatedly, receiving an HIV diagnosis caused a range of emotions, from indifference to sadness. Overwhelmingly the reactions were negative. The most common was sadness about having to live with HIV (n=8). Participants also mentioned experiencing depressive symptoms, which stemmed from their belief that HIV was a “death sentence” (n=5). The reactions differed by gender, such that women all noted that they were sad, devastated, or depressed by the news. Whereas some men did mention shock, denial, and depression, others expressed feeling

as if were in “limbo” about their ability to work and maintain their livelihoods. Specifically, they worried if they would continue to have the physical strength to maintain their job.

Management. At the time of the interview, all participants stated that they were engaged in medication taking and treatment. Although several noted not immediately taking medications because they felt fine when diagnosed or because they initially had adverse reactions to the medications. Participants mentioned that they could live a normal life with HIV as long as they took their medications, went to the doctor, practiced safe sexual practices, and took care of their nutritional needs. The automation of self-management led participants to not overly think or worry about their HIV (*no dar mente*), which was important because several participants linked the rumination over their HIV status with the development of depressive symptoms. A few participants explicitly mentioned that having access to government social services (e.g., subsidized medication, food supplement program) also enabled them to manage their condition. There were two primary challenges participants faced when managing their HIV. The first was stigma, which men spoke more often about, specifically judgement from others who held inaccurate beliefs about HIV. Persons holding stigmatizing beliefs could be family, friends, neighbors, and colleagues, which complicated who they could receive social support from. Second, both men and women spoke equally about the related difficulty of maintaining partners as they experienced rejections and judgments from potential partners once participants disclosed their HIV status.

Living with a non-communicable chronic condition

Diagnosis. Most participants were diagnosed with a chronic NCD at a clinic or hospital. Several participants (n=8) were diagnosed when they had to go to a health care facility because they felt ill, such as feeling dizzy, excessively tired, “suffocated”, or experienced chest pains.

Several participants (n=5) noted that they were diagnosed with a chronic condition during a HIV treatment follow-up at a clinic. For these participants their only healthcare provider was their HIV provider, who may have offered dietary and physical activity guidance. Participant responses to their diagnosis were primarily negative. Sadness and fear were the most common reactions as participants cited knowing family or friends who had passed away from a related condition (e.g., T2D, hypertension). Relatedly, inevitability was another reaction particularly if there were several family members who also lived with the condition.

Management. At the time of the interview, only three participants were able to follow-up on their respective diagnosis with a chronic condition-specific provider. Implications of those who could not follow-up with providers meant that participants were delayed in establishing a treatment plan to address the specific condition. As one participant, a 53-year-old man with T2D and hypertension, explained his difficulties with getting a formal diagnosis affected his ability to begin treatment:

> Every time I come to the [HIV] clinic my blood sugar is high, but I have yet to get a diagnosis so I can't see a specialist to see if I need medication or not, because at this time I am not taking anything.

For those who were diagnosed and linked to care, they often spoke of needing support to manage their medications and key behaviors, primarily diet and physical activity. Five facilitators that participants described as helping them self-manage their chronic conditions were: reduced medication costs; dietary education; improved social support from friends and family; referrals or access to specialists, and maintaining a “calm” demeanor. Of the five facilitators, the notion of maintaining a calm demeanor as a practice came from participants with either hypertension or T2D. For participants with hypertension, emotional regulation was tied to the belief that blood pressure is sensitive to emotions. For those with T2D, the explanation was that T2D caused

individuals to be “hot blooded”, a stigmatizing belief; therefore, they needed to consciously work on regulating their emotions for their health.

Living with the double burden of conditions

Participants explained that HIV had its challenges with management; however, there was a consensus among all participants that managing a chronic NCD was more difficult for them. A few participants specifically mentioned that they had trouble getting their chronic NCDs “under control”. For example, a couple participants with hypertension described how they had to seek care at a clinic or emergency room rather recently due to blood pressure dysregulation, and therefore found a condition like hypertension to be much more unpredictable and difficult to manage than HIV. The perception of several participants when comparing HIV to another condition was that more people were dying from conditions such as T2D complications than HIV, making chronic NCDs more dangerous to live with, as mentioned by a 44-year-old man living with high cholesterol: “At the moment, I would say that HIV is easier to manage because I have medical care [for it] here at the hospital.”

Even without the long-term view, there was a perception that T2D was worse than HIV because it would inevitably “spoil” your body from inside out, as described by a 41-year-old woman living with T2D:

> With HIV you can live for some time when you take your medication, but with “sugar” it spoils you from the inside.

Conversely, participants believed that they could “live a normal life” with HIV if they took their ARTs consistently. There was some discussion of eating healthier and taking care of themselves in general, but all participants emphasized medication adherence as the primary form of HIV management. When participants described their experiences with certain chronic conditions, such as T2D and hypertension, medication use was also noted as an important

component of self-management; however, medication use was not the only concern. The primary concern was that participants felt burdened by having to change their lifestyles. As one 52-year-old woman managing obesity described of T2D, “You cannot eat anything. You cannot eat much rice, and you cannot eat spaghetti.” This was further expounded upon by a 56-year-old woman managing hypertension and T2D when explaining which condition was worse for her:

> [Diabetes] because with HIV I take my pill and I feel fine. But with diabetes sometimes when one eats something their blood sugar immediately goes up. I must stay vigilant that I can’t eat this or can’t eat that. That’s why it’s more worrisome.

There were concerns about T2D-related complications such as vision loss and amputations. Similarly, hypertension caused fear because people could “die instantly” from one moment to the next. Overall, there was a feeling of not having as much control over these conditions as compared to HIV, where all participants felt relatively secure that HIV-related complications were no longer a threat to their well-being.

Participants provided examples of how their experiences co-managing these conditions varied at the individual, interpersonal, and structural level. A summary of these experiences is provided in Table 2 and in further detail in subsequent sections.

Table 2. Multi-level Participant Experiences with Co-managing Conditions

	HIV	Chronic Conditions (Non-communicable)
**Self-management**	Generally, participants described that it was easier to manage HIV due to:<br>• Better knowledge (“orientation”), practice, self-efficacy, and consistent access to medications to self-manage their HIV	Consensus was that there were more difficulties to self-manage their chronic conditions for several reasons:<br>• The unpredictability of the daily changes of their conditions, such as daily blood pressure spiking or

	• Primary behavior for HIV self-management was consistent medication taking	blood glucose increasing or decreasing<br>• Need for more "orientation" or knowledge on how to best self-manage<br>• Medication taking was inconsistent due to cost, ability to get (re)fills<br>• Several chronic conditions are sensitive to behaviors other than medication, such as diet, which was difficult to always do consistently
**Patient-provider**	• All participants had built rapport with their HIV providers and mentioned having good patient-provider interactions<br>o Providers described as friendly, available (can answer questions after hours), and supportive<br>• Most participants named their HIV providers as their primary care doctor<br>• HIV providers did some basic follow-up that was relevant to specific conditions, particularly hypertension; however, it was not comprehensive care	• Most participants did not have a regular provider to address their chronic condition<br>• Rapport building with providers was limited due to inconsistency in attending different places
**Access to Care**	• Referral system once diagnosed with HIV was clear, therefore linkage to care was consistent<br>o Participants felt that it was easier to get linked to care for HIV<br>• Participants attributed consistent HIV care with low or no cost for health appointments	• Several participants were diagnosed with T2D or had elevated blood glucose (i.e., prediabetes) but were not referred to a specialist<br>• Several participants used emergency room services to address any health emergencies (hypertension, T2D)<br>• Specialist care can be cost-prohibitive making it hard to establish care, build rapport, and have consistent follow-up to evaluate changes to the condition<br>• A few participants did not know where to seek care to address their conditions<br>• There were a range of care options participants used for chronic disease management

		○ Government clinics (*policlinica*) when needed<br>○ Went to specialists when funds were available for clinic fees or for transportation (many specialists were in larger cities)<br>○ Asked HIV providers if they have any general questions

Individual level: Self-management

As participants spoke of their self-management of multiple chronic conditions, there were distinct reasons that self-management was either straightforward or challenging. HIV self-management was most often described as straightforward primarily for two reasons. The first was that participants believed that they had sufficient knowledge or "orientation" about what HIV was and what they needed to do to manage the condition, and they had enough time to practice the skills they needed to self-manage, which was related to the improved self-efficacy. Second, all participants mentioned that the consistent access to necessary medications facilitated their ability to adhere to their treatment plan.

On the other end, participants found self-management of their chronic NCDs to be more difficult. Unlike the amount of orientation they received for HIV, participants felt that they received limited education on how to best self-manage their specific chronic condition. The lack of education on NCD self-management was also tied to how participants experienced their condition as being "sensitive" and "unpredictable" from one day to the next. This is exemplified by a 48-year-old woman with T2D and hypertension.

> I manage HIV more easily than diabetes because with HIV I take my medication just fine. But with diabetes, even with I take the medication, sometimes based on what I eat, my blood sugar goes up […] Also with diabetes, if you cut yourself

> the cut can start to rot – it's the worst disease. They say that HIV is bad, but the worst are cancer and diabetes.

As described above, hypertension and T2D were cited as being difficult as they were both very sensitive to dietary habits, and were prone to unpredictable extremes (e.g., hyperglycemia, uncontrolled high blood pressure) that would incapacitate participants or require them to seek emergency care.

Interpersonal level: Patient-provider interactions

Interpersonal relationships were an important theme from the interviews. All participants described that they had "good" interactions with their HIV providers. Specifically, HIV providers were described as friendly, responsive at all times of the day, and supportive, as explained by a 34-year-old man managing obesity when speaking about his providers:

> [The doctors] are always looking after you, and they have what I would say – rather it makes me feel good. Because the [doctors] support us, they are always with us.

These positive-leaning relationships with their providers were noteworthy because most participants named their HIV providers as their primary care provider (*doctor de cabecera*). Participants described the range of services they received from their HIV providers, for example, they received standard HIV-related care, HIV providers evaluated their HIV medication adherence, and followed up on their progress living with HIV.

Only two participants stated that they had more than one medical provider. In addition to their HIV providers, both participants had access to a cardiologist to help them manage their hypertension. One participant explained that they met with their cardiologist on a consistent

basis, whereas the second participant met with theirs on an inconsistent basis due to financial constraints related to paying the consultation fee. Of note, participants explained that neither provider discussed the co-management of both of their conditions.

Structural level: Access to care

Both individual self-management and interpersonal interactions with providers are dependent on facilitators and barriers at the structural level. Structurally, a third of the study participants did not have health insurance. Two participants lost coverage between 6-month and 12-month follow-ups, however, their narratives regarding care seeking did not seem to change based on their change in health insurance status. Of the five participants who were without health insurance throughout the 12-month study period, three were the participants of Haitian descent. Of the 14 participants with health insurance, only three participants had a medical provider for each chronic condition. Overwhelmingly, participants with health insurance noted not being linked to care for their chronic NCD due to accessibility barriers. Thus, the narratives between participants with and without health insurance were similar.

The narratives on access to health care were distinct for HIV as compared to chronic NCDs. According to participants, the HIV system was clearer or “easier.” Specifically, once participants were diagnosed with HIV, they all described getting linked to the appropriate hospital/clinic and healthcare team. Once linked to the appropriate HIV specialist, participants stated that they also were able to receive low or no cost consultations and medications. Participants attributed their consistent HIV self-management to the financial support they received that facilitated their health care access.

For their chronic NCD condition, the narratives were varied due to inconsistent referrals and poor or no financial assistance. This comparison was detailed by a 49-year-old man managing hypertension and cholesterol.

> HIV is easier because I just need to have transportation money to come here and do not need to pay for anything else. But for blood pressure, for cardio, I must always bring money because it's a private clinic.

First, participants did not present narratives detailing a consistent referral system after diagnosis. Several participants who were diagnosed with T2D or had elevated blood sugar (i.e., prediabetes) were not referred to specialists upon diagnosis. Further, a few participants with hypertension spoke about not having access to a specialist for their care.

When asked if participants knew where to seek care for their conditions the responses were either they did not know, the emergency room, or they spoke with their HIV providers. Emergency rooms were often stated as the primary site to address health needs as they arose and could be their primary source of information for managing their condition. This was especially the case for participants who described living too far away from specialists or never receiving a medical referral. This balance was explained by 53-year-old man living with hypertension and T2D:

> They send us to Santiago. There is not a clinic here that you could say, let me go there because it is for diabetes or something. I think that for emergencies if you go to the emergency room and a doctor sees you and finds out that you have high blood pressure or some other problem, then you will get referred to see a specialist.

When it came to asking their HIV providers for help or follow-up on non-HIV conditions, the most common example was that HIV providers checked their blood pressure thus participants

deemed these vital sign checks as part of their care for hypertension. As for financial resources to seek care, participants mentioned that they could only go to specialists when they could save enough money for the transportation to the appointment, the consult, and any prescribed medications. Relatedly, participants spoke about how job insecurity and limited financial resources made it harder to seek specialized care and/or purchase necessary medications. The issue of the cost of medication was highlighted by a 48-year-old woman living with hypertension and T2D:

> Treatment for diabetes is expensive. So, the diabetes treatment is not given to people. See, when I came here the doctor prescribed me a pill that cost me 4,000 and some pesos. Tell me, people who are poor and do not work may be able to buy one box of medication, but would not be able to buy two?

The above participant quote highlighted the dilemmas faces by people who have limited resources when managing their chronic conditions. For participants that could not afford consistent access to specialists, a couple would go to government clinics (*policlínicas*) when needed for basic checkups.

The inconsistency of health care access, whether due to unclear referral system to establish care or lack of financial resources to see and build rapport with specialists, made it challenging for participants to manage their chronic condition. Structural barriers to care affected individual and interpersonal level factors impacting the co-management of multiple chronic conditions.

Discussion

Adults experiencing food insecurity in the Dominican Republic who manage HIV and at least one chronic NCD, encounter multi-level barriers and facilitators for appropriate co-

management. Understanding these multi-level factors can improve our ability to develop appropriate programs and policies to improve the management of multiple conditions among adults.

Health insurance coverage and its utility came through in participant narratives. Participants without health insurance did not seek care outside of the HIV clinic. Of note, all three Haitian-descendent participants did not have health insurance, like reflecting their limited access to health insurance programs given the sociopolitical barriers this community faces such as the high cost of applying or renewing documentation (e.g., Dominican identification card), which is needed to apply for government insurance coverage [37, 38]. Participants who had health insurance noted the same structural barriers to seeking care, primarily the physical distance to seek specialists and the high out-of-pocket costs. Relatedly, participants spoke of the need to “save money” to seek care for non-HIV specialists or delay care due to the high costs of transportation, consults, and medication. The expectations of high out-of-pocket costs for health care was evidenced in the narratives of balancing financial needs and health care needs, particularly for chronic NCDs. This is reflective in a 2016 report in which the out-of-pocket costs at the point of service averaged at about 44.6% of the total cost of care in the Dominican Republic, which is at the higher end of the Americas region which averages 23.9% [39].

Additionally, access to medications was a fundamental barrier for self-management among participants. Medication affordability as a factor to engaging in chronic disease management also came up in a cross-sectional study by Castro and colleagues [8] who found that affordability of medications was a primary concern for Dominican adults living with hypertension or T2D. Cost concerns were not as large of an issue for HIV because the Dominican government provides all HIV medications and clinical care at no charge However, similar programs are not available for chronic NCDs. Since 2001, laws were passed to promote

universal health coverage in the public health sectors, which included preventive care and outpatient drug coverage. In relation to the health system, in 2006 the National Public Health Services (SENASA) constructed a network of subsidized private health providers that could be affiliated with the public health network to improve financial accessibility to care [40]. In terms of outpatient drug coverage, this program covers RD$8,000 pesos (USD$142) per year [41]; however, because of the high costs of medication, the coverage may only last two or three months. In 2008, a plan was introduced to integrate chronic disease programs into the primary care model [8]. Shortly thereafter in 2009, the Ministry of Public Health developed the National Non-Communicable Chronic Disease Control and Prevention Program, aimed at the prevention and management of chronic NCDs and any associated risk factors [8]. In October 2015 an interinstitutional agreement was signed by CONAVIHSIDA, which is the National Council for HIV and AIDS, SENASA, and the Central Electoral Council to fund improved access to the public health system for PLHIV [42]. The implications of this agreement were such that it allowed for PLHIV to request care for their HIV and other chronic conditions regardless of documentation status. The results of this are indicated in how participants described their ease of access to care for their HIV. These evolutions of the health system since the turn of the century indicates that health systems are responsive to the changing health priorities. However, participant narratives showed that barriers remain on the implementation of improved care for their chronic NCDs. For instance, participants often cited their HIV clinician as their primary care provider, thus there were limited interactions for participants in primary care settings as intended by the 2008 program.

Participants spoke often about not receiving or not being aware of a referral to see a specialist for their condition or having their HIV provider offer follow-up for their non-HIV conditions. Contextually, these practice differences may be due to the capacities of the HIV program centers. There are some centers that have internal medicine doctors who can provide

general follow-up to their HIV and other comorbidities; however, they will refer more severe cases to specialists. This may explain why some participants noted that their HIV provider managed all aspects of their care. Other centers do not have more general medical practitioners available, therefore they only refer their patients to specialists. On the referral side, there is no system where HIV providers can communicate with specialists to follow-up with them regarding the status of any referred patients. The different methods used based on center capacity and the barriers to following up on referrals, may have contributed to participant confusion on how to gain access to other forms of specialized care for their chronic NCDs. Improved standardization of the referral process and increased clarity of the health care process during patient visits may help HIV center patients better navigate managing their health. In addition, these barriers suggest perhaps integrating the care of chronic NCDs within HIV care settings as a possible way of improving coordination between specialties, reducing barriers for patients seeking care, and improved holistic management for adults with multiple chronic conditions.

In terms of patient-provider relationships, all participants detailed a positive and productive relationship with their HIV providers. Although there was a lack of clear referrals for specialists to manage their chronic NCDs, additional barriers may emerge for PLHIV when seeking care. A 2018 scoping review among PLHIV seeking to manage chronic NCDs found that fear of disclosure, internalized shame, negative past experiences with healthcare providers, or negative perceptions of healthcare providers, as well as HIV stigma from the part of providers all affected engagement in health care [22]. Therefore, work must be done ensure that healthcare providers are trained on empathetic care and work to change any stigmatizing attitudes and perceptions they may have towards future patients who live with HIV, to ensure that patients are not confronted with hostile environments.

Living a normal life was the most salient goal expressed by all participants. A qualitative study in the Dominican Republic found that the concept of “living a normal life” as related to HIV

was indeed related to the notion of HIV becoming a “chronic condition” [43]. Nevertheless, in Barrington et al. [43], achieving a normal life was complicated by HIV-related stigma and discrimination, implications for social relationships, and economic productivity, particularly for individuals living in resource-constrained areas. We found parallels in our study related to living a “normal life” with HIV. “Normal” in this case (for adults experiencing food insecurity) was attributed to their ability to consistently engage in treatment. In other words, the idea that they could live a long life with HIV equated to living a normal life. However, participants did speak about fears related to economic loss (e.g., not being able to work again) and rejection they faced from potential partners when they disclosed their HIV status, thus complicating their ability to develop social ties. In relation to chronic NCDs, striving to live a normal life was not brought up as consistently. This may have been due to the uncertainty around of the management of chronic NCDs, which added a barrier to achieving the goal of living a normal life. Furthermore, there were parallels between how adults with HIV in our study and adult with T2D in the Dominican Republic in a recent qualitative study described the difficulties associated with chronic conditions. Specifically, there was a perception that T2D was the worse condition compared to HIV because T2D required a multi-component self-management strategy that upended their entire lives, requiring not only medications, but also drastic changes in diet, and additional physical activity [30].

T2D was also considered a worse condition because of the stigmatizing stereotype attached to people with T2D, that they are quick to anger or get agitated (*pique*). Therefore, the need for people living with T2D to engage in emotional regulation was not only for their health but to counteract stereotypes. Concerns about the stereotypes associated with people with T2D were evident in other qualitative studies conducted in the Dominican Republic [30, 44]. When addressing interventions or programs that include people with T2D and HIV, ensuring that programs address the social stigmas associated with both conditions are necessary.

Strengths and limitations

The strengths of this study are that we explored the multilevel factors that affect co-managing multiple chronic conditions from the perspective of adults in the Dominican Republic, to our knowledge one of the first of its kind. Additionally, we focused our study on participants who were experiencing food insecurity, which centers the conversation on individuals who are managing their health amid economic vulnerabilities. Limitations of the study were that we selected and interviewed participants who were already linked to HIV care and were at the end of an intervention; therefore, the experiences of individuals who were currently not in HIV-related care were not reflected in these interviews. However, participants were able to reflect on their experiences getting linked to care, retrospectively, to provide a baseline comparison between their conditions.

Conclusions

This study depicts the facilitators and barriers of managing multiple chronic conditions from the perspective of adults experiencing food insecurity in the Dominican Republic. As the number of adults living with HIV and other chronic conditions are increasing, we must contextualize these experiences to build future programs or policies that improve the health and well-being of individuals, particularly those who a navigating economic and social vulnerabilities. Individual self-management is necessary; however, it is not sufficient for the successful co-management of multiple chronic conditions. Interactions or relationships participants had with their health care team, improved referral systems, accessibility and availability of specialists, and financial support systems are important dimensions to assess in the development of health systems that account for the management of multiple chronic conditions.

Acknowledgements

The authors thank the participants for their generosity in providing their time as well as the health clinic staff and providers that participated. The authors would also like to thank Dr. Victor Terrero, Director of CONVIHSIDA for their ongoing support for our efforts to understand and address food insecurity among people living with HIV. We thank colleagues from the Universidad Autónoma de Santo Domingo UASD and Ministry of Public Health Nutrition.

References

1. UNAIDS. Latin American Region Switzerland: The Joint United Nations Programme on HIV/AIDS (UNAIDS); 2021a [cited 2021 August 20]. Available from: https://www.unaids.org/en/regionscountries/latinamerica
2. Avert. United Kindgom: Avert; 2020 [cited 2021 August 20]. Available from: https://www.avert.org/professionals/hiv-around-world/latin-america/overview
3. UNAIDS. Dominican Republic 2020: Country factsheets Switzerland: The Joint United Nations Programme on HIV/AIDS (UNAIDS); 2021b [cited 2021 August 20]. Available from: https://www.unaids.org/en/regionscountries/countries/dominicanrepublic
4. PEPFAR. Dominican Republic - Country Operational Plan (COP/ROP) 2019: Strategic Direction Summary. Washington, DC: US President's Emergency Plan for AIDS Relief, 2019 April 1, 2019. Report No.
5. CDC. Global Health - Dominican Republic Atlanta, Georgia: Centers for Disease Control and Prevention; 2019 [cited 2021 August 20]. Available from: https://www.cdc.gov/globalhealth/countries/dr/default.htm
6. van Olmen J, Schellevis F, Van Damme W, Kegels G, Rasschaert F. Management of chronic diseases in Sub-Saharan Africa: Cross-fertilisation between HIV/AIDS and diabetes care. Journal of Tropical Medicine. 2012;2012:349312. doi: 10.1155/2012/349312.

7. WHO. Global Health Estimates: Life expectancy and leading causes of death and disability Geneva, Switzerland: World Health Organization; 2021 [cited 2021 September 7]. Available from: https://www.who.int/data/gho/data/themes/mortality-and-global-health-estimates.
8. Castro B, Ing L, Park Y, Abrams J, Ryan M. Addressing noncommunicable disease in Dominican Republic: Barriers to hypertension and diabetes care. Annals of Global Health. 2018;84(4):625. doi: 10.29024/aogh.2370.
9. World Bank. Causes of death, by non-communicable diseases (% of total) - Dominican Republic Geneva, Switzerland: World Bank; 2020 [cited 2021 August 23]. Available from: https://data.worldbank.org/indicator/SH.DTH.NCOM.ZS?locations=DO.
10. Sherman BW. Management of individuals with multiple chronic conditions: A continuing challenge. The American Journal of Managed Care. 2021;27(6):256-60. doi: 10.37765/ajmc.2021.88665.
11. DHHS. Multiple chronic conditions - a strategic framework: Optimum health and quality of life for individuals with multiple chronic conditions. Washington, DC: US Department of Health and Human Services, 2010 December 2010. Report No.
12. Basu J, Avila R, Ricciardi R. Hospital readmission rates in U.S. states: Are readmissions higher where more patients with multiple chronic conditions cluster? Health Services Research. 2016;51(3):1135-51. doi: 10.1111/1475-6773.12401.
13. Ippolito MM, Lyles CR, Prendergast K, Marshall MB, Waxman E, Seligman HK. Food insecurity and diabetes self-management among food pantry clients. Public Health Nutrition. 2017;20(1):183-9. Epub 2016/07/13. doi: 10.1017/S1368980016001786.
14. Schwartz SR, Kavanagh MM, Sugarman J, Solomon SS, Njindam IM, Rebe K, et al. HIV viral load monitoring among key populations in low- and middle-income countries: challenges and opportunities. Journal of the International AIDS Society. 2017;20. doi: 10.1002/jia2.25003.

15. Mattei J, Tamez M, Ríos-Bedoya CF, Xiao RS, Tucker KL, Rodríguez-Orengo JF. Health conditions and lifestyle risk factors of adults living in Puerto Rico: A cross-sectional study. BMC Public Health. 2018;18(1):491. doi: 10.1186/s12889-018-5359-z.
16. Chen W-T, Wantland D, Reid P, Corless IB, Eller LS, Iipinge S, et al. Engagement with Health Care Providers Affects Self- Efficacy, Self-Esteem, Medication Adherence and Quality of Life in People Living with HIV. Journal of AIDS & Clinical Research. 2013;4(11):256. doi: 10.4172/2155-6113.1000256.
17. Abebe F, Schneider M, Asrat B, Ambaw F. Multimorbidity of chronic non-communicable diseases in low- and middle-income countries: A scoping review. Journal of Comorbidity. 2020;10:2235042X20961919. doi: 10.1177/2235042X20961919.
18. Tsegaye M, Chelkeba L, Mekonnen Z, Kumela K. Glycemic Control Among People Living with Diabetes and Human Immunodeficiency Virus in Ethiopia: Leveraging Clinical Care for the Looming Co-Epidemics. Diabetes, Metabolic Syndrome and Obesity. 2020;13:4379-99. doi: 10.2147/DMSO.S266105. PubMed PMID: 2461079088.
19. Mitra S, Atrouni WE, Olotu B, Rasu R. Diabetes, mortality, and the burden of comorbidities in people living with HIV (PLHIV), and the effect of exposure to protease inhibitors: A single-center study in the midwestern United States. Open Forum Infectious Diseases. 2016;3(suppl_1). doi: 10.1093/ofid/ofw172.1689.
20. Colasanti J, Galaviz KI, Mehta CC, Palar K. Room for improvement: The HIV-diabetes care continuum over 15 years in the women's interagency HIV study. Open Forum Infectious Diseases. 2018;5(6). doi: 10.1093/ofid/ofy121.
21. Costa CRB, Melo ES, Antonini M, Pontes PS, Gir E, Reis RK. Sobrepeso e obesidade em pessoas que vivem com HIV. Revista Gaúcha de Enfermagem. 2021;42.
22. Stockton MA, Kayla G, Nyblade L. A scoping review of the role of HIV-related stigma and discrimination in noncommunicable disease care. PLoS One. 2018;13(6). doi: 10.1371/journal.pone.0199602. PubMed PMID: 2057883476.

23. Bigna JJ, Ndoadoumgue AL, Nansseu JR, Tochie JN. Global burden of hypertension among people living with HIV in the era of increased life expectancy: a systematic review and meta-analysis. Journal of Hypertension. 2020;38(9):1659-68. doi: 10.1097/HJH.0000000000002446.
24. Derose KP, Payán DD, Fulcar MA, Terrero S, Acevedo R, Farías H, et al. Factors contributing to food insecurity among women living with HIV in the Dominican Republic: A qualitative study. PLoS One. 2017;12(7). doi: 10.1371/journal.pone.0181568. PubMed PMID: 1923274353.
25. Payán DD, Derose KP, Fulcar MA, Farías H, Palar K. “It was as though my spirit left, like they killed me”: The disruptive impact of an HIV-positive diagnosis among women in the Dominican Republic. Journal of the International Association of Providers of AIDS Care (JIAPAC). 2019;18:2325958219849042. doi: 10.1177/2325958219849042.
26. Brooks K, Laws MB, Wilson I. Allocating HIV treatment to the adherent: A qualitative study of patient perceptions of their HIV care in the Dominican Republic. Journal of Health Care for the Poor and Underserved. 2014;25(4):1748-62. doi: 10.1353/hpu.2014.0190.
27. Kerrigan D, Barrington C, Donastorg Y, Perez M, Galai N. Abriendo Puertas: Feasibility and effectiveness a multi-level intervention to improve HIV outcomes among female sex workers living with HIV in the Dominican Republic. AIDS and Behavior. 2016;20(9):1919-27. doi: 10.1007/s10461-016-1376-z.
28. Madsen Beau De Rochars VE, Keys H, Samuels SK, Jo A, Noland GS, Gonzales M, et al. Prevalence of Diabetes, Prediabetes, and Associated Risk Factors Among Agricultural Village Residents in the Dominican Republic. Am J Trop Med Hyg. 2021. doi: 10.4269/ajtmh.19-0942. PubMed PMID: 33872205.
29. Sadeghzadeh C, Wallace DD, Gonzalez Rodriguez H, Barrington C. Coping with diabetes stress among adults in rural Dominican Republic:“I don’t think about it”. Chronic Illness. 2019:1742395319882069. doi: 10.1177/1742395319882069.

30. Gonzalez Rodriguez H, Wallace DD, Barrington C. Contextualizing experiences of diabetes-related stress in rural Dominican Republic. Qualitative Health Research. 2019;29(6):857-67. doi: 10.1177/1049732318807207.

31. Weiser SD, Young SL, Cohen CR, Kushel MB. Conceptual framework for understanding the bidirectional links between food insecurity and HIV/AIDS. The American Journal of Clinical Nutrition. 94(6):1729S-39S. doi: 10.3945/ajcn.111.012070.

32. Gucciardi E, Vahabi M, Norris N, Del Monte JP, Farnum C. The intersection between food insecurity and diabetes: A review. Current nutrition reports. 2014;3(4):324-32. Epub 2014/11/11. doi: 10.1007/s13668-014-0104-4. PubMed PMID: 25383254; PubMed Central PMCID: PMCPMC4218969.

33. Derose KP, Fulcar MA, Acevedo R, Armenta G, Jiménez-Paulino G, Bernard CL, et al. An integrated urban gardens and peer nutritional counseling intervention to address food insecurity among people with HIV in the Dominican Republic. AIDS Education and Prevention. 2021;33(3):187-201. doi: 10.1521/aeap.2021.33.3.187.

34. Devers KJ, Frankel RM. Study design in qualitative research--2: Sampling and data collection strategies. Education for Health 2000;13(2):263-71. Epub 2004/01/27. doi: 10.1080/13576280050074543. PubMed PMID: 14742088.

35. Braun V, Clarke V. Using thematic analysis in psychology. Qualitative Research in Psychology. 2006;3(2):77-101. doi: 10.1191/1478088706qp063oa. PubMed PMID: 223135521.

36. Dedoose. Web application for managing, analyzing, and presenting qualitative and mixed method research data. Version 8.0.35 ed. Los Angeles, CA: SocioCultural Research Consultants, LLC; 2018.

37. Leventhal S. A gap between ideals and reality: The right to health and the inaccessibility of healthcare for Haitian migrant workers in the Dominican Republic. Emory International Law Review. 2013;27(2).

38. Keys HM, Keys HM, Kaiser BN, Foster JW, Burgos Minaya RY. Perceived discrimination, humiliation, and mental health: A mixed-methods study among Haitian migrants in the Dominican Republic. Ethnicity & Health. 2015;20(3):219-40. doi: 10.1080/13557858.2014.907389.

39. PAHO. Indicadores básicos 2019: Tendencias de la salud en las Américas. Washington, DC: Pan American Health Organization, 2019 October 1, 2019. Report No.

40. Ministerio de Salud Pública (MSP). Plan nacional de prevencion y control de las enfermedades no transmisibles 2019-2024. Santo Domingo, Republic Dominicana Ministerio de Salud Pública y Asistencia Social, 2019.

41. Ministerio de Salud Pública (MSP). Republic Dominicana - Plan estrategico control integrado ECNT Santo Domingo, Republica Dominicana2014 [cited 2020 October 21]. Available from: https://www.iccp-portal.org/republica-dominicana-plan-estrategico-control-integrado-ecnt.

42. Seguro National de Salud. SeNaSA, el SNS, JCE, y CONAVIHSIDA firman acuerdo interinstitucional para afiliar al régimen subsidiado personas que viven con VIH Santo Domingo, Republica Dominicana: Seguro Nacional de Salud (SENASA); 2019 [cited 2021 October 21]. Available from: https://www.arssenasa.gob.do/index.php/blog/2019/10/15/senasa-el-sns-jce-y-conavihsida-firman-acuerdo-interinstitucional-para-afiliar-al-regimen-subsidiado-personas-que-viven-con-vih/.

43. Barrington C, Kerrigan D, Ureña FIC, Brudney K. La vida normal: living with HIV in Santo Domingo, Dominican Republic. Culture, Health & Sexuality. 2018;20(1):40-54. doi: 10.1080/13691058.2017.1323350.

44. Wallace DD, Gonzalez Rodriguez H, Walker E, Dethlefs H, Dowd RA, Filipi L, et al. Types and sources of social support among adults living with type 2 diabetes in rural communities

in the Dominican Republic. Global Public Health. 2019;14(1):135-46. doi: 10.1080/17441692.2018.1444782.

Click here to access/download
Supporting Information
PLOS_Questionnaire_Inclusivity.docx

The co-management of HIV and chronic non-communicable diseases in the Dominican Republic: A qualitative study

Deshira D. Wallace[1*¶], Amarilis Then-Paulino[2&], Gipsy Jiménez Paulino[3&], Flabia Tejada Castro[4&], Stephanie Daniela Castro[5&], Kartika Palar[6&], Kathryn P. Derose[7,8¶]

[1]Department of Health Behavior, Gillings School of Global Public Health, University of North Carolina at Chapel Hill, Chapel Hill, North Carolina, United States of America

[2]Escuela de Salud Pública, Facultad de Ciencias de la Salud, Universidad Autónoma de Santo Domingo, Alma Máter, Ciudad Universitaria, Santo Domingo, Dominican Republic

[3]Viceministerio de Garantía de la Calidad, Ministerio de Salud Pública, Santo Domingo, Dominican Republic

[4]Independent Consultant, Santo Domingo, Dominican Republic

[5]Center for Diagnosis, Advanced Medicine, and Telemedicine (CEDIMAT), Santo Domingo, Dominican Republic

[6]Department of Medicine, Division of HIV, Infectious Disease, and Global Medicine, University of California, San Francisco, San Francisco, California, United States of America

[7]Department of Health Promotion & Policy, University of Massachusetts Amherst, Amherst, Massachusetts, United States of America

[8]Department of Behavioral and Policy Sciences, RAND Corporation, Santa Monica, California, United States of America

*Corresponding author

Email: ddwallac@email.unc.edu (DDW)

[¶]These authors contributed equally to this work.

[&]These authors also contributed equally to this work.

Abstract

People living with HIV and a non-communicable disease (NCD) experience multi-level barriers when co-managing both conditions. We explored the factors affecting living with multiple chronic conditions in the Dominican Republic.

We conducted 21 in-depth interviews from October 2019-February 2020 with Dominican adults experiencing food insecurity and managing HIV and at least one chronic NCD. Using thematic analysis, we explored participant lived experiences co-managing multiple chronic conditions.

All participants (mean age = 45.5 years) were linked to HIV care, but only three were linked to NCD-specific care. Individual-level barriers to managing NCDs included limited ~~self-management education~~education and limited self-efficacy for self-management.~~, and~~ I~~i~~nterpersonally, barriers included limited rapport building with an NCD-specific specialist. Structurally, barriers to managing NCDs were no health insurance, poor referral systems, and limited financial assistance.

Health system adaptation requires equitably considering the needs of individuals managing multiple chronic conditions. Key factors to address include patient-provider relationships, improved referral systems, accessibility and availability of specialists, and financial assistance.

Introduction

The Caribbean region has been undergoing a health transition in which traditional infectious diseases have been occurring alongside with a rise of chronic non-communicable diseases (NCDs). As of 2019 approximately 1.1% of people in the Caribbean are living with HIV, making it the region with the second highest HIV prevalence after sub-Saharan Africa [1, 2]. Within the Caribbean, the Dominican Republic has a similarly high HIV prevalence comparable to the region at approximately 0.9% of the population [3, 4]. In fact, HIV/AIDS-related complications are the fifth cause of death in the country [5]. Of the individuals living with HIV in the Dominican Republic, 82% know their status; further, ~~and~~ 51% of people living HIV, ~~regardless of status,~~ are on antiretroviral therapy (ART) [3]. Although HIV is an infectious disease, advances in treatment have shifted HIV to function more as a chronic condition, requiring similar behaviors for its management such as medication adherence, lifestyle adjustments, consistent and long-term provider interaction, and adequate interpersonal support [6].

As related to chronic NCDs, diabetes mellitus and cardiovascular diseases are among the leading causes of death and morbidity globally [7]. Chronic NCDs include a range of conditions such as cardiovascular diseases, type 2 diabetes mellitus (T2D), cancer, pulmonary diseases, neurological conditions, and mental health conditions. Trends in the global rise of chronic NCDs are similar in the Dominican Republic [8]. Cardiovascular diseases (ischemic heart disease, intracerebral hemorrhage, ischemic stroke) are three of the four top causes of death in the Dominican Republic [5]. Overall chronic NCDs accounted for 72% of deaths in the Dominican Republic in 2019, a 40% increase since 2010 [9].

With HIV being managed as a chronic condition, and the continued rise of chronic NCDs, there is acknowledgement that these conditions do not happen in silos. Thus, a shift in models of care that account for the management of multiple chronic conditions is necessary.

However, as individuals with multiple chronic conditions represent an increasing proportion of the adult population worldwide, studies have found that multilevel barriers affect the effectiveness of care for these individuals [10, 11]. At the individual level, those with multiple chronic conditions require greater healthcare utilization and therefore incur greater healthcare costs than individuals with one or zero chronic conditions [12]. The costs associated with high utilization can stem from emergency room use, inpatient hospitalizations, outpatient consultations and care, and medication use [10]. The cost of managing HIV and chronic NCDs disproportionately affect individuals who are socially and economically marginalized. Specifically, self-management for persons experiencing intersecting marginalized social and economic conditions, such as food insecurity, require using a significantly higher proportion of their available income compared to individuals who are economically secure and do not experience various social forms of marginalization and stigma [13, 14]. Interpersonally, individuals with multiple chronic conditions require sufficient social support from family, friends, and their healthcare team (i.e., patient-provider relationships) to manage their care [15, 16]. At the structural level, ~~including~~ the lack of coordination between primary care providers and specialists, poor coordination among specialists to ensure suggested treatments are aligned, and healthcare accessibility~~,~~ (both distance and financial), and availability are important for the management of each condition [10]. These barriers are further complicated in low-and-middle income countries (LMICs) where the health systems are focused on addressing burdens of communicable diseases such as vector-borne conditions and HIV, as well as maternal, child and neonatal health [17]. Studies from Ethiopia and the United States have found an increasing number of people living with HIV (PLHIV) are not meeting glycemic control targets [18-20]. A study among 340 PLHIV in Brazil found a significant number of respondents were ~~managing~~ dealing with obesity, high cholesterol, and a significantly higher likelihood of metabolic syndrome [21]. A recent meta-analysis estimated that the regional prevalence of hypertension among PLHIV in Latin America and the Caribbean at 22% (17.8-26.5) indicating the increasingly

overlapping health needs of PLHIV [22, 23]. Therefore, accounting for individual, interpersonal, and structural facilitators and barriers to disease co-management is needed when evaluating living with multiple chronic conditions.

Although there are rich data on the experiences of managing HIV across several sub-populations in the Dominican Republic (e.g., urban versu~~e~~s rural, by occupation) [24-27], and there are increasing numbers of studies on ~~type 2 diabetes (~~T2D~~)~~ and cardiovascular diseases in the Dominican Republic [28-30]; to our knowledge, there is little data on the co-management of these conditions, particularly in a country with substantial investment in HIV care while balancing the rising burden of NCDs. Furthermore, ~~as~~ food insecurity has been shown to affect management of both HIV and chronic NCDs such as T2D.~~,~~ In fact, food insecurity ~~affects~~ ~~poor~~adversely affects HIV outcomes and NCDs through a variety of behavioral, nutritional, and psychosocial pathways [31, 32]. Understanding the realities of adults in structurally vulnerable food insecurity states, can improve our understanding of ways to ~~improve~~adapt systems to improve ~~multiple disease~~disease co-~~-~~management for broader segments of the population. Therefore, we were interested in exploring how adults experiencing food insecurity, and thus living in more resource-constrained environments, are managing multiple conditions in the Dominican Republic.

We use the socioecological model to inform this study, particularly in the exploration of the multidimensional nature of facilitators and barriers to living with and managing multiple chronic conditions in urbanized areas in the Dominican Republic. Specifically, we conducted qualitative interviews to explore the lived experiences of adults in the Dominican Republic who are experiencing food insecurity and managing both HIV and at least one chronic NCD and constructed themes in relation to individual level, interpersonal level, and structural level factors affecting their health and wellbeing. The contribution of this work is that it provides formative

information on the considerations of public health approaches to address multiple condition management in a Dominican or similar contexts.

Materials and methods

Study

This qualitative study is part of an endline evaluation of a 12-month pilot intervention led by researchers at the RAND Corporation, a research non-profit organization, and the School of Health Sciences at the Universidad Autónoma de Santo Domingo (UASD). The intervention (i.e., ProMeSA) integrated urban gardens and peer nutritional counseling for adults with HIV experiencing moderate or severe food insecurity and treatment adherence difficulties in urban centers in the Dominican Republic [33]. The intervention comprised gardening training, home-based agricultural follow-up, peer nutritional counseling, and a group garden-based cooking workshop. The participants underwent two follow-up periods to assess potential changes HIV clinical outcomes (viral load, ART adherence, and HIV care retention) and other HIV-related outcomes (internalized and experienced stigma, social support). in skills and knowledge over time.

The endline qualitative study involved in-depth interviews with patients participating in the pilot intervention program at the intervention clinic (another clinic served as a usual care control). The broader goal of the interviews involved exploring health care experiences, facilitators and barriers to managing HIV, facilitators and barriers to managing a chronic NCD, and participants' perceptions regarding the feasibility, acceptability, and impacts of the intervention. This article focuses on the facilitators and barriers of co-managing HIV and a chronic NCD.

Setting and participants

The intervention study, ProMeSA ~~study,~~ was conducted at two HIV clinics located in the central and northwestern part of the Dominican Republic [33]. The clinics are approximately a 2-hour drive from each other to avoid cross-contamination between participants. Both clinics were selected as they were of similar sizes 500-800 patients on ART and offered similar standards of care. Participants in the intervention study were 18 years and older, registered patient at the HIV clinic sites, resided in an urban or peri-urban area, had moderate or severe food insecurity, and had evidence of adherence difficulties. For the qualitative endline sub-study, we used in-person purposive sampling [34] to select intervention participants who also had a chronic NCD (i.e., hypertension, diabetes, prediabetes, high cholesterol, obesity) according to previously collected data. We purposively recruited approximately equal numbers of men and women. Furthermore, participants were recruited until we reached thematic saturation. Institutional Review Boards and ethics committees from RAND, UASD, and the Dominican Ministry of Public Health approved the study protocols. All intervention study participants provided written consent; for the additional qualitative endline interview, participants provided ~~oral~~ verbal consent to account for literacy and accessibility.

Data collection

Three Dominican medical school graduates (one as intervention study coordinator [GJP] and two as research assistants [FTC, SDC]) were trained in qualitative data collection and analysis by KD and DW prior to and throughout the study period. This involved virtual and in-person trainings in Santo Domingo on how to conduct semi-structured interviews, manage data, transcribe audio recordings, and code transcripts. We used semi-structured, in-depth interviews to explore our themes of interest for this study: health experiences living with HIV, health experiences living with a chronic NCD, and health services experiences with both conditions.

Using a socioecological model, we framed questions to understand individual, interpersonal, organizational, and political barriers and facilitators to managing multiple chronic conditions. Example questions included: how are you managing your HIV? How are you managing your chronic condition (T2D~~type 2 diabetes~~, high blood pressure)? Which of your health conditions worries you the most?

Qualitative interviews were completed between October 2019 – February 2020 at the ~~two HIV~~ intervention clinic~~s~~ in the Dominican Republic. All interviews were conducted in Spanish, lasted between 45-90 minutes, and were audio recorded with participant consent. After each interview, interviewers wrote field notes about the interview setting, key themes of the interview, observations for the following interviews, and if any themes may have reached saturation. Audio recordings were transcribed verbatim by the three trained interviewers/research assistants and verified by the first author.

Data analysis

We used a thematic analysis approach to explore participants' lived experiences co-managing multiple health conditions through the examination of deductive and inductive codes [35]. To do that, we first uploaded transcripts to Dedoose, a web-based text management and analysis software [36]. We then developed a codebook, which was initially developed using questions from the interview guide and field note observations. The codebook was iteratively updated based on the coding of initial transcripts. Four trained coders (DW, GJP, FTC, DC) read and coded the transcripts independently and the first author conducted a secondary coding review. Transcripts coded by the first author were reviewed by another member of the coding team. When reading the transcripts, coders wrote memos, or notes, about their observations of the data and linked what they read with other transcripts. All coding and analyses were

completed in Spanish. Any discrepancies or questions about coding were resolved via coder consensus.

Some key codes we used to analyze participant experiences were: how participants were diagnosed with HIV and the chronic NCD, emotional reactions to their diagnoses, their understanding/knowledge of either condition, how they managed their conditions, social support with management, and their experiences with the healthcare system for both conditions. After coding, all codes were placed in a matrix organized by participant to facilitate constant comparison of experiences among participants and within codes. We constructed summaries by code to examine similarities and differences in participant experiences, and then combined codes to construct overarching themes. Themes are organized using a socioecological framework approach, with consideration for individual, interpersonal, and structural facilitators and barriers to disease co-management in the Dominican Republic.

Results

A total of 21 participants were recruited, of which 12 were men and 9 were women. The mean age of participants was 45.5 years old. Eighteen participants self-identified as being of Dominican descent and three self-identified as being of Haitian descent. While all participants were diagnosed with HIV, participants had a range of chronic NCDs. These conditions included type 2 diabetesT2D, hypertension, high cholesterol, and cardiovascular and circulatory diseases (see Table 1). Note that individuals could be listed for multiple chronic NCDs.

Table 1. Participant demographics (n=21)

Variable	N	%	Mean
Age (years)			45.5
Female	9	42.9	
Ancestry			
Dominican	18	85.7	

Haitian	3	14.3	
Active Insurance Coverage	14	66.7	
Non-Communicable Chronic Conditions			
Prediabetes	5	23.8	
Diabetes	5	23.8	
Overweight/Obesity	12	57.1	
Hypertension	7	33.3	
High Cholesterol	3	14.3	
Cardiovascular disease	1	4.8	
Thrombosis	1	4.8	
Number of Chronic Conditions			
1	10	47.6	
2	7	33.3	
3	4	19.0	

The results are organized by providing a narrative of HIV management, narrative of chronic NCD management, and how these conditions compare in terms of their ability to co-manage them at the individual, interpersonal, and structural levels. Since only individuals who had experienced moderate or severe food insecurity were enrolled in the intervention study, all narratives are filtered through those experiences.

Living with HIV

Diagnosis. Participant narratives all began at diagnosis. All but three participants were diagnosed at a clinic or hospital, and the remaining three had a workplace screening that triggered a clinic referral. The reasons for initial HIV testing varied by gender with women describing getting tested for two reasons. The first was getting tested because their partner or spouse received a positive test, had HIV-related complications, or passed away due to untreated HIV complications. The second reason was pregnancy as two women tested positive after their second trimester tests. In contrast, men often got tested because they fell sick and went to a clinic where they learned their status and a few men went in for regular HIV/STI testing and ~~got~~ received their diagnosi~~e~~s~~d~~ then.

Relatedly, receiving an HIV diagnosis caused a range of emotions, from indifference to sadness. Overwhelmingly the reactions were negative. The most common was sadness about having to live with HIV (n=8). Participants also mentioned experiencing depressive symptoms, which stemmed from their belief that HIV was a “death sentence” (n=5). The reactions differed by gender, such that women all noted that they were sad, devastated, or depressed by the news. Whereas some men did mention shock, denial, and depression, others expressed feeling as if were in “limbo” about their ability to work and maintain their livelihoods. Specifically, they worried if they would continue to have the physical strength to maintain their job.

Management. At the time of the interview, all participants stated that they were engaged in medication taking and treatment.~~,~~ ~~a~~Although several noted not immediately taking medications because they felt fine when diagnosed or because they initially ~~having~~ had adverse reactions to the medications. Participants mentioned that they could live a normal life with HIV as long as they took their medications, went to the doctor, practiced safe sexual practices, and took care of their nutritional needs. The automation of self-management led participants to not overly think or worry about their HIV (*no dar mente*), which was important because several participants linked the rumination over their HIV status with the development of depressive symptoms. A few participants explicitly mentioned that having access to government social services (e.g., subsidized medication, food supplement program) also enabled them to manage their condition. There were two primary challenges participants faced when managing their HIV. The first was stigma, which men spoke more often about, specifically judgement from others who held inaccurate beliefs about HIV. Persons holding stigmatizing beliefs could be family, friends, neighbors, and colleagues, which complicated who they could receive social support from. Second, both men and women spoke equally about the related difficulty of maintaining partners as they experienced rejections and judgments from potential partners once participants disclosed their HIV status.

Living with a non-communicable chronic condition

Diagnosis. Most participants were diagnosed with a chronic NCD at a clinic or hospital. Several participants (n=8) were diagnosed when they had to go to a health care facility because they felt ill, such as feeling dizzy, excessively tired, “suffocated”, or experienced chest pains. Several participants (n=5) noted that they were diagnosed with a chronic condition during a HIV treatment follow-up at a clinic. For these participants their only healthcare provider was their HIV provider, who may have offered dietary and physical activity guidance. Participant responses to their diagnosis were primarily negative. Sadness and fear were the most common reactions as participants cited knowing family or friends who had passed away from a related condition (e.g., T2D, hypertension). Relatedly, inevitability was another reaction particularly if there were several family members who also lived with the condition.

Management. At the time of the interview, only three participants were able to follow-up on their respective diagnosis with a chronic condition-specific provider. Implications of those who could not follow-up with providers meant that participants were delayed in establishing a treatment plan to address the specific condition. As one participant, a 53-year-old man with T2D and hypertension, explained his difficulties with getting a formal diagnosis affected ~~their~~ his ability to begin treatment:

> Every time I come to the [HIV] clinic my blood sugar is high, but I have yet to get a diagnosis so I can’t see a specialist to see if I need medication or not, because at this time I am not taking anything.

For those who were diagnosed and linked to care, they often spoke of needing support to manage their medications and key behaviors, primarily diet and physical activity. Five facilitators that participants described ~~needing to~~as helping them self-manage their chronic conditions

were: reduced medication costs; dietary education; improved social support from friends and family; referrals or access to specialists, and maintaining a “calm” demeanor. Of the five facilitators, the notion of maintaining a calm demeanor as a practice came from participants with either hypertension or T2D. For participants with hypertension, emotional regulation was tied to the belief that blood pressure is sensitive to emotions. For those with T2D, the explanation was that T2D caused individuals to be “hot blooded”, a stigmatizing belief; therefore, they needed to consciously work on regulating their emotions for their health.

Living with the double burden of conditions

Participants explained that HIV had its challenges with management; however, there was a consensus among all participants that managing a chronic NCD was more difficult for them. A few participants specifically mentioned that they had trouble getting their chronic NCDs “under control”. For example, a couple participants with hypertension described how they had to seek care at a clinic or emergency room rather recently due to blood pressure dysregulation, and therefore found a condition like hypertension to be much more unpredictable and difficult to manage than HIV. The perception of several participants when comparing HIV to another condition was that more people were dying from conditions such as T2D complications than HIV, making chronic NCDs more dangerous to live with, as mentioned by a 44-year-old man living with high cholesterol: “At the moment, I would say that HIV is easier to manage because I have medical ~~attention~~ care [for it] here at the hospital.”

Even without the long-term view, there was a perception that T2D was worse than HIV because it would inevitably “spoil” your body from inside out, as described by a 41-year-old woman living with T2D:

> With HIV you can live for some time when you take your medication, but with “sugar” it spoils you from the inside.

Conversely, participants believed that they could “live a normal life” with HIV if they took their ARTs consistently. There was some discussion of eating healthier and taking care of themselves in general, but all participants emphasized medication adherence as the primary form of HIV management. When participants described their experiences with certain chronic conditions, such as T2D and hypertension, medication use was also noted as an important component of self-management; however, medication use was not the only concern. The primary concern was ~~they~~that participants felt burdened by having to change their lifestyles. As one 52-year-old woman managing obesity described of T2D, “You cannot eat anything. You cannot eat much rice, and you cannot eat spaghetti.”~~.~~ This was further expounded upon by a 56-year-old woman managing hypertension and T2D when explaining which condition was worse for her:

> [Diabetes] because with HIV I take my pill and I feel fine. But with diabetes sometimes when one eats something their blood sugar immediately goes up. I must stay vigilant that I can’t eat this or can’t eat that. That’s why it’s more worrisome.

There were concerns about T2D-related complications such as vision loss and amputations. Similarly, hypertension caused fear because people could “die instantly” from one moment to the next. Overall, there was a feeling of not having as much control over these conditions as compared to HIV, where all participants felt relatively secure that HIV-related complications were no longer a threat to their well-being.

Participants provided examples of how their experiences co-managing these conditions varied at the individual, interpersonal, and structural level. A summary of these experiences is provided in Table 2 and in further detail in subsequent sections.

Table 2. Multi-level Participant Experiences with Co-managing Conditions

	HIV	Chronic Conditions (Non-communicable)
**Self-management**	Generally, participants described that it was easier to manage HIV due to:<br>• ~~Participants felt that they had~~Better ~~a~~ knowledge (“orientation”), practice, self-efficacy, and consistent access to medications to self-manage their HIV<br>• Primary behavior for HIV self-management was consistent medication taking	Consensus was that there were more difficulties to self-manage their chronic conditions for several reasons:<br>• The unpredictability of the daily changes of their conditions, such as daily blood pressure spiking or blood glucose increasing or decreasing<br>• Need for more “orientation” or knowledge on how to best self-manage<br>• Medication taking was~~is~~ inconsistent due to cost, ability to get (re)fills<br>• Several chronic conditions are sensitive to behaviors other than medication, such as diet, which was difficult to always do consistently
**Patient-provider**	• All participants had built rapport with their HIV providers and mentioned having good patient-provider interactions<br>○ Providers described as friendly, available (can answer questions after hours), and supportive<br>• Most participants named their HIV providers as their primary care doctor<br>• HIV providers ~~may do~~did some basic follow-up that was~~is~~ relevant to specific conditions, particularly hypertension; however, it was~~is~~ not comprehensive care	• Most participants did not have a regular provider to address their chronic condition<br>• Rapport building with providers was limited due to inconsistency in attending different places
**Access to Care**	• Referral system once diagnosed with HIV was clear, therefore linkage to care was consistent<br>○ Participants felt that it was easier to get linked to care for HIV<br>• Participants attributed consistent HIV care with low or no cost for health appointments	• Several participants were diagnosed with ~~type 2 diabetes~~T2D or had elevated blood glucose (i.e., prediabetes) but were not referred to a specialist<br>• Several participants used emergency room services to address any health emergencies (hypertension, T2D)

		• Specialist care can be cost-prohibitive making it hard to establish care, build rapport, and have consistent follow-up to evaluate changes to the condition<br>• A few participants did not know where to seek care to address their conditions<br>• There ~~was~~ were a range of care options participants used for chronic disease management<br>○ Government clinics (*policlinica*) when needed<br>○ Went~~Go~~ to specialists when funds were available for clinic fees or for transportation (many specialists were in larger cities)<br>○ ~~May ask~~Asked HIV providers if they have any general questions

Individual level: Self-management

As participants spoke of their self-management of multiple chronic conditions, there were distinct reasons that self-management was either straightforward or challenging. HIV self-management was most often described as straightforward primarily for two reasons. The first was that participants believed that they had sufficient knowledge or "orientation" about what HIV was and what they needed to do to manage the condition, and they had enough time to practice the skills they needed to self-manage, which was related to the improved self-efficacy. Second, all participants mentioned that the consistent access to necessary medications facilitated their ability to adhere to their treatment plan.

On the other end, participants found self-management of their chronic NCDs to be more difficult. Unlike the amount of orientation~~,~~ they received for HIV, participants felt that they received limited education on how to best self-manage their specific chronic condition. The lack

of education on NCD self-management was also tied to how participants experienced their condition as being “sensitive” and “unpredictable” from one day to the next. This is exemplified by a 48-year-old woman with T2D and hypertension.

> I manage HIV more easily than diabetes because with HIV I take my medication just fine. But with diabetes, even with I take the medication, sometimes based on what I eat, my blood sugar goes up […] Also with diabetes, if you cut yourself the cut can start to rot – it’s the worst disease. They say that HIV is bad, but the worst are cancer and diabetes.

As described above, hypertension and T2D were cited as being difficult as they were both very sensitive to dietary habits, and were prone to unpredictable extremes (e.g., hyperglycemia, uncontrolled high blood pressure) that would incapacitate participants or require them to seek emergency care.

Interpersonal level: Patient-provider interactions

Interpersonal relationships were an important theme from the interviews. All participants described that they had “good” interactions with their HIV providers. Specifically, HIV providers were described as friendly, responsive at all times of the day, and supportive, as explained by a 34-year-old man managing obesity when speaking about his providers:

> [The doctors] are always looking after you, and they have what I would say – rather it makes me feel good. Because the [doctors] support us, they are always with us.

These positive-leaning relationships with their providers were noteworthy because most participants named their HIV providers as their primary care provider (*doctor de cabecera*).

Participants described the range of services they received from their HIV providers, for example, they received standard HIV-related care, HIV providers evaluated their HIV medication adherence, and followed up on their progress living with HIV.

Only two participants stated that they had more than one medical provider. In addition to their HIV providers, both participants had access to a cardiologist to help them manage their hypertension. One participant explained that they met with their cardiologist on a consistent basis, whereas the second participant met with theirs on an inconsistent basis due to financial constraints related to paying the consultation fee. Of note, participants explained that neither provider discussed the co-management of both of their conditions.

Structural level: Access to care

Both individual self-management and interpersonal interactions with providers are dependent on facilitators and barriers at the structural level. Structurally, a third of the study participants did not have health insurance. Two participants lost coverage between 6-month and 12-month follow-ups, however, their narratives regarding care seeking did not seem to change based on their change in health insurance status. Of the five participants who were without health insurance throughout the 12-month study period, three were the participants of Haitian descent. Of the 14 participants with health insurance, only three participants had a medical provider for each chronic condition. Overwhelmingly, participants with health insurance noted not being linked to care for their chronic NCD due to accessibility barriers. Thus, the narratives between participants with and without health insurance were similar.

The narratives on access to health care were distinct for HIV as compared to chronic NCDs. According to participants, the HIV system was clearer or “easier.”~~.~~ Specifically, once participants were diagnosed with HIV, they all described getting linked to the appropriate

hospital/clinic and health-care team. Once linked to the appropriate HIV specialist, participants stated that they also were able to receive low or no cost consultations and medications. Participants attributed their consistent HIV self-management to the financial support they received that facilitated their health care access.

For their chronic NCD condition, the narratives were varied due to inconsistent referrals and poor or no financial assistance. This comparison was detailed by a 49-year-old man managing hypertension and cholesterol.

> HIV is easier because I just must need to have transportation money to come here and do not need to pay for anything else. But for blood pressure, for cardio, I must always bring money because it's a private clinic.

First, participants did not present narratives detailing a consistent referral system after diagnosis. Several participants who were diagnosed with T2D or had elevated blood sugar (i.e., prediabetes) were not referred to specialists upon diagnosis. Further, a few participants with hypertension spoke about not having access to a specialist for their care.

When asked if participants knew where to seek care for their conditions the responses were either they did not know, the emergency room, or they spoke with their HIV providers. Emergency rooms were often stated as the primary site to address health needs as they arose and could be their primary source of information for managing their condition. This was especially the case for participants who noted that theydescribed livinged too far away from specialists or never receivinged a medical referral. This balance was explained by 53-year-old man living with hypertension and T2D:

> They send us to Santiago. There is not a clinic here that you could say, let me go there because it is for diabetes or something. I think that for emergencies if you go to the emergency room and a doctor sees you and finds out that you have

> high blood pressure or some other problem, then you will get referred to see a specialist.

When it came to asking their HIV providers for help or follow-up on non-HIV conditions, the most common example was that HIV providers checked their blood pressure thus participants deemed these vital sign checks as part of their care for hypertension. As for financial resources to seek care, participants mentioned that they could only go to specialists when they could save enough money for the transportation to the appointment, the consult, and any prescribed medications. Relatedly, participants spoke about how job insecurity and limited financial resources made it harder to seek specialized care and/or purchase necessary medications. The issue of the cost of medication was highlighted by a 48-year-old woman living with hypertension and T2D:

> Treatment for diabetes is expensive. So, the diabetes treatment is not given to people. See, when I came here the doctor prescribed me a pill that cost me 4,000 and some pesos. Tell me, people who are poor and do not work may be able to buy one box of medication, but would not be able to buy two?

The above participant quote highlighted the dilemmas faces by people who have limited resources when managing their chronic conditions. For participants that could not afford consistent access to specialists, a couple would go to government clinics (*policlínicas*) when needed for basic checkups.

The inconsistency of health care access, whether due to unclear referral system to establish care or lack of financial resources to see and build rapport with specialists, made it challenging for participants to manage their chronic condition. Structural barriers to care affected individual and interpersonal level factors impacting the co-management of multiple chronic conditions.

Discussion

Adults experiencing food insecurity in the Dominican Republic who manage HIV and at least one chronic NCD, encounter multi-level barriers and facilitators for appropriate co-management. Understanding these multi-level factors can improve our ability to develop appropriate programs and policies to improve the management of multiple conditions among adults.

Health insurance coverage and its utility came through in participant narratives. Participants without health insurance did not seek care outside of the HIV clinic. Of note, all three Haitian-descendent participants did not have health insurance, like reflecting ~~which brings into question~~ their limited access to health insurance programs given the sociopolitical barriers this ~~population~~ community faces such as the high cost of applying or renewing documentation (e.g., Dominican identification card), which is needed to apply for government insurance coverage [37, 38]. Participants who had health insurance noted the same structural barriers to seeking care, primarily the physical distance to seek specialists and the high out-of-pocket costs. Relatedly, participants spoke of the need to “save money” to seek care for non-HIV specialists or delay care due to the high costs of transportation, consults, and medication. The expectations of high out-of-pocket costs for health care was evidenced in the narratives of balancing financial needs and health care needs, particularly for chronic NCDs. This is reflective in a 2016 report in which the out-of-pocket costs at the point of service averaged at about 44.6% of the total cost of care in the Dominican Republic, which is at the higher end of the Americas region which averages 23.9% [39].

Additionally, access to medications was a fundamental barrier for self-management among participants. Medication affordability as a factor to engaging in chronic disease

management also came up in a cross-sectional study by Castro and colleagues [8] who found that affordability of medications was ~~also~~ a primary concern for Dominican adults living with hypertension or T2D. Cost concerns were not as large of an issue for HIV ~~due to the fact that~~because the Dominican government provides all HIV medications and clinical care ~~free~~ at~~of~~ no charge However, similar programs are not available for chronic NCDs. Since 2001, laws were passed to promote universal health coverage in the public health sectors, which included preventive care and outpatient drug coverage. In relation to the health system, in 2006 the National Public Health Services (SENASA) constructed a network of subsidized private health providers that could be affiliated with the public health network to improve financial accessibility to care [40]. In terms of outpatient drug coverage, this program covers RD$8,000 pesos (USD$142) per year [41]; however, because of the high costs of medication, the coverage may only last two or three months. In 2008, a plan was introduced to integrate chronic disease programs into the primary care model [8]. Shortly thereafter in 2009, the Ministry of Public Health developed the National Non-Communicable Chronic Disease Control and Prevention Program, aimed at the prevention and management of chronic NCDs and any associated risk factors [8]. In October 2015 an interinstitutional agreement was signed ~~between~~ by CONAVIHSIDA, which is the National Council for HIV and AIDS, SENASA, and the Central Electoral Council to fund improved access to the public health system for PLHIV [42]. The implications of this agreement were such that it allowed for PLHIV to request care for their HIV and other chronic conditions regardless of documentation status. The results of this are indicated in how participants described their ease of access to care for their HIV. These evolutions of the health system since the turn of the century indicates that health systems are~~is~~ responsive to the changing health priorities. However, participant narratives showed that barriers remain on the implementation of improved care for their chronic NCDs. For instance, participants often cited their HIV clinician as their primary care provider, thus there ~~was~~ were limited interactions for participants in primary care settings as intended by the 2008 program.

Participants spoke often about not receiving or not being aware of a referral to see a specialist for their condition or having their HIV provider offer follow-up for their non-HIV conditions. Contextually, these practice differences may be due to the capacities of the HIV program centers. There are some centers that have internal medicine doctors who can provide general follow-up to their HIV and other comorbidities; however, they will refer more severe cases to specialists. This may explain why some participants noted that their HIV provider managed all aspects of their care. Other centers do not have more general medical practitioners available, therefore they only refer their patients to specialists. On the referral side, there is no system where HIV providers can communicate with specialists to follow-up with them regarding the status of any referred patients. The different methods used based on center capacity and the barriers to following up on referrals, may have contributed to participant confusion on how to gain access to other forms of specialized care for their chronic NCDs. Improved standardization of the referral process and increased clarity of the health care process during patient visits may help HIV center patients better navigate managing their health. In addition, these barriers suggest perhaps integrating the care of chronic NCDs within HIV care settings as a possible way of improving coordination between specialties, reducing barriers for patients seeking care, and improved holistic management for adults with multiple chronic conditions.

In terms of patient-provider relationships, all participants detailed a positive and productive relationship with their HIV providers. Although there was a lack of clear referrals for specialists to manage their chronic NCDs, additional barriers may emerge for PLHIV when seeking care. A 2018 scoping review among PLHIV seeking to manage chronic NCDs found that fear of disclosure, internalized shame, negative past experiences with healthcare providers, or negative perceptions of healthcare providers, as well as HIV stigma from the part of providers all affected engagement in health care [22]. Therefore, work must be done ensure that healthcare providers are trained on empathetic care and work to change any stigmatizing

attitudes and perceptions they may have towards future patients who live with HIV, to ensure that patients are not confronted with hostile environments.

Living a normal life was the most salient goal expressed by all participants. A qualitative study in the Dominican Republic found that the concept of “living a normal life” as related to HIV was indeed related to the notion of HIV becoming a “chronic condition” [43]. Nevertheless, in Barrington et al. [43], ~~the strive to living a~~achieving a normal life was complicated by HIV-related stigma and discrimination, ~~and~~ implications for social relationships, and economic productivity, particularly for individuals living in resource-constrained areas. We found parallels in our study related to living a “normal life” with HIV. “Normal” in this case (for adults experiencing food insecurity)~~,~~ was attributed to their ability to consistently engage in treatment. In other words, the idea that they could live a long life with HIV equated to living a normal life. However, participants did speak about fears related to economic loss (e.g., not being able to work again) and rejection they faced from potential partners when they disclosed their HIV status, thus complicating their ability to develop social ties. In relation to chronic NCDs, striving ~~the strive~~ to live a normal life was not brought up as consistently. This may have been due to the uncertainty around of the management of chronic NCDs, which added a barrier to achieving the goal of living a normal life. Furthermore, there were parallels between how adults with HIV in our study and adult with T2D in the Dominican Republic in a recent qualitative study described the difficulties associated with chronic conditions. Specifically, there was a perception that T2D was the worse condition compared to HIV because T2D required a multi-component self-management strategy that upended their entire lives, requiring not only medications, but also drastic changes in diet, and additional physical activity [30].

T2D was also considered a worse condition because of the stigmatizing stereotype attached to people with T2D, that they are quick to anger or get agitated (*pique*). Therefore, the need for people living with T2D to engage in emotional regulation was not only for their health

but to counteract stereotypes. Concerns about the stereotypes associated with people with T2D were evident in other qualitative studies conducted in the Dominican Republic [30, 44]. When addressing interventions or programs that include people with T2D and HIV, ensuring that programs address the social stigmas associated with both conditions are necessary.

Strengths and limitations

The strengths of this study are that we explored the multilevel factors that affect co-managing multiple chronic conditions from the perspective of adults in the Dominican Republic, to our knowledge one of the first of its kind. Additionally, we focused ~~on~~ our study on participants who were experiencing food insecurity, which centers the conversation on individuals who are managing their health amid economic vulnerabilities. Limitations of the study were that we selected and interviewed participants who were already linked to HIV care and were at the end of an intervention; therefore, the experiences of individuals who were currently not in HIV-related care were not reflected in these interviews. However, participants were able to reflect on their experiences getting linked to care, retrospectively, to provide a baseline comparison between their conditions.

Conclusions

This study depicts the facilitators and barriers of managing multiple chronic conditions ~~for~~from the perspective of adults experiencing food insecurity in the Dominican Republic. As the number of adults living with HIV and other chronic conditions are increasing, we must contextualize ~~those~~these experiences to build future programs or policies that improve the health and well-being of individuals, particularly those who a navigating economic and social vulnerabilities. Individual self-management is necessary; however, it is not sufficient for the successful co-management of multiple chronic conditions. Interactions or relationships

participants had with their health care providers, or health team, improved referral systems, accessibility and availability of specialists, and financial support systems are important dimensions to assess in the development of health systems that account for the management of multiple chronic conditions.

Acknowledgements

The authors thank the participants for their generosity in providing their time as well as the health clinic staff and providers that participated. The authors would also like to thank Dr. Victor Terrero, Director of CONVIHSIDA for their ongoing support for our efforts to understand and address food insecurity among people living with HIV. We thank colleagues from the Universidad Autónoma de Santo Domingo UASD and Ministry of Public Health Nutrition.

References

1. UNAIDS. Latin American Region Switzerland: The Joint United Nations Programme on HIV/AIDS (UNAIDS); 2021a [cited 2021 August 20]. Available from: https://www.unaids.org/en/regionscountries/latinamerica
2. Avert. United Kindgom: Avert; 2020 [cited 2021 August 20]. Available from: https://www.avert.org/professionals/hiv-around-world/latin-america/overview
3. UNAIDS. Dominican Republic 2020: Country factsheets Switzerland: The Joint United Nations Programme on HIV/AIDS (UNAIDS); 2021b [cited 2021 August 20]. Available from: https://www.unaids.org/en/regionscountries/countries/dominicanrepublic
4. PEPFAR. Dominican Republic - Country Operational Plan (COP/ROP) 2019: Strategic Direction Summary. Washington, DC: US President's Emergency Plan for AIDS Relief, 2019 April 1, 2019. Report No.

5. CDC. Global Health - Dominican Republic Atlanta, Georgia: Centers for Disease Control and Prevention; 2019 [cited 2021 August 20]. Available from: https://www.cdc.gov/globalhealth/countries/dr/default.htm
6. van Olmen J, Schellevis F, Van Damme W, Kegels G, Rasschaert F. Management of chronic diseases in Sub-Saharan Africa: Cross-fertilisation between HIV/AIDS and diabetes care. Journal of Tropical Medicine. 2012;2012:349312. doi: 10.1155/2012/349312.
7. WHO. Global Health Estimates: Life expectancy and leading causes of death and disability Geneva, Switzerland: World Health Organization; 2021 [cited 2021 September 7]. Available from: https://www.who.int/data/gho/data/themes/mortality-and-global-health-estimates.
8. Castro B, Ing L, Park Y, Abrams J, Ryan M. Addressing noncommunicable disease in Dominican Republic: Barriers to hypertension and diabetes care. Annals of Global Health. 2018;84(4):625. doi: 10.29024/aogh.2370.
9. World Bank. Causes of death, by non-communicable diseases (% of total) - Dominican Republic Geneva, Switzerland: World Bank; 2020 [cited 2021 August 23]. Available from: https://data.worldbank.org/indicator/SH.DTH.NCOM.ZS?locations=DO.
10. Sherman BW. Management of individuals with multiple chronic conditions: A continuing challenge. The American Journal of Managed Care. 2021;27(6):256-60. doi: 10.37765/ajmc.2021.88665.
11. DHHS. Multiple chronic conditions - a strategic framework: Optimum health and quality of life for individuals with multiple chronic conditions. Washington, DC: US Department of Health and Human Services, 2010 December 2010. Report No.
12. Basu J, Avila R, Ricciardi R. Hospital readmission rates in U.S. states: Are readmissions higher where more patients with multiple chronic conditions cluster? Health Services Research. 2016;51(3):1135-51. doi: 10.1111/1475-6773.12401.

13. Ippolito MM, Lyles CR, Prendergast K, Marshall MB, Waxman E, Seligman HK. Food insecurity and diabetes self-management among food pantry clients. Public Health Nutrition. 2017;20(1):183-9. Epub 2016/07/13. doi: 10.1017/S1368980016001786.

14. Schwartz SR, Kavanagh MM, Sugarman J, Solomon SS, Njindam IM, Rebe K, et al. HIV viral load monitoring among key populations in low- and middle-income countries: challenges and opportunities. Journal of the International AIDS Society. 2017;20. doi: 10.1002/jia2.25003.

15. Mattei J, Tamez M, Ríos-Bedoya CF, Xiao RS, Tucker KL, Rodríguez-Orengo JF. Health conditions and lifestyle risk factors of adults living in Puerto Rico: A cross-sectional study. BMC Public Health. 2018;18(1):491. doi: 10.1186/s12889-018-5359-z.

16. Chen W-T, Wantland D, Reid P, Corless IB, Eller LS, Iipinge S, et al. Engagement with Health Care Providers Affects Self- Efficacy, Self-Esteem, Medication Adherence and Quality of Life in People Living with HIV. Journal of AIDS & Clinical Research. 2013;4(11):256. doi: 10.4172/2155-6113.1000256.

17. Abebe F, Schneider M, Asrat B, Ambaw F. Multimorbidity of chronic non-communicable diseases in low- and middle-income countries: A scoping review. Journal of Comorbidity. 2020;10:2235042X20961919. doi: 10.1177/2235042X20961919.

18. Tsegaye M, Chelkeba L, Mekonnen Z, Kumela K. Glycemic Control Among People Living with Diabetes and Human Immunodeficiency Virus in Ethiopia: Leveraging Clinical Care for the Looming Co-Epidemics. Diabetes, Metabolic Syndrome and Obesity. 2020;13:4379-99. doi: 10.2147/DMSO.S266105. PubMed PMID: 2461079088.

19. Mitra S, Atrouni WE, Olotu B, Rasu R. Diabetes, mortality, and the burden of comorbidities in people living with HIV (PLHIV), and the effect of exposure to protease inhibitors: A single-center study in the midwestern United States. Open Forum Infectious Diseases. 2016;3(suppl_1). doi: 10.1093/ofid/ofw172.1689.

20. Colasanti J, Galaviz KI, Mehta CC, Palar K. Room for improvement: The HIV-diabetes care continuum over 15 years in the women's interagency HIV study. Open Forum Infectious Diseases. 2018;5(6). doi: 10.1093/ofid/ofy121.

21. Costa CRB, Melo ES, Antonini M, Pontes PS, Gir E, Reis RK. Sobrepeso e obesidade em pessoas que vivem com HIV. Revista Gaúcha de Enfermagem. 2021;42.

22. Stockton MA, Kayla G, Nyblade L. A scoping review of the role of HIV-related stigma and discrimination in noncommunicable disease care. PLoS One. 2018;13(6). doi: 10.1371/journal.pone.0199602. PubMed PMID: 2057883476.

23. Bigna JJ, Ndoadoumgue AL, Nansseu JR, Tochie JN. Global burden of hypertension among people living with HIV in the era of increased life expectancy: a systematic review and meta-analysis. Journal of Hypertension. 2020;38(9):1659-68. doi: 10.1097/HJH.0000000000002446.

24. Derose KP, Payán DD, Fulcar MA, Terrero S, Acevedo R, Farías H, et al. Factors contributing to food insecurity among women living with HIV in the Dominican Republic: A qualitative study. PLoS One. 2017;12(7). doi: 10.1371/journal.pone.0181568. PubMed PMID: 1923274353.

25. Payán DD, Derose KP, Fulcar MA, Farías H, Palar K. "It was as though my spirit left, like they killed me": The disruptive impact of an HIV-positive diagnosis among women in the Dominican Republic. Journal of the International Association of Providers of AIDS Care (JIAPAC). 2019;18:2325958219849042. doi: 10.1177/2325958219849042.

26. Brooks K, Laws MB, Wilson I. Allocating HIV treatment to the adherent: A qualitative study of patient perceptions of their HIV care in the Dominican Republic. Journal of Health Care for the Poor and Underserved. 2014;25(4):1748-62. doi: 10.1353/hpu.2014.0190.

27. Kerrigan D, Barrington C, Donastorg Y, Perez M, Galai N. Abriendo Puertas: Feasibility and effectiveness a multi-level intervention to improve HIV outcomes among female sex workers

living with HIV in the Dominican Republic. AIDS and Behavior. 2016;20(9):1919-27. doi: 10.1007/s10461-016-1376-z.

28. Madsen Beau De Rochars VE, Keys H, Samuels SK, Jo A, Noland GS, Gonzales M, et al. Prevalence of Diabetes, Prediabetes, and Associated Risk Factors Among Agricultural Village Residents in the Dominican Republic. Am J Trop Med Hyg. 2021. doi: 10.4269/ajtmh.19-0942. PubMed PMID: 33872205.
29. Sadeghzadeh C, Wallace DD, Gonzalez Rodriguez H, Barrington C. Coping with diabetes stress among adults in rural Dominican Republic:“I don’t think about it”. Chronic Illness. 2019:1742395319882069. doi: 10.1177/1742395319882069.
30. Gonzalez Rodriguez H, Wallace DD, Barrington C. Contextualizing experiences of diabetes-related stress in rural Dominican Republic. Qualitative Health Research. 2019;29(6):857-67. doi: 10.1177/1049732318807207.
31. Weiser SD, Young SL, Cohen CR, Kushel MB. Conceptual framework for understanding the bidirectional links between food insecurity and HIV/AIDS. The American Journal of Clinical Nutrition. 94(6):1729S-39S. doi: 10.3945/ajcn.111.012070.
32. Gucciardi E, Vahabi M, Norris N, Del Monte JP, Farnum C. The intersection between food insecurity and diabetes: A review. Current nutrition reports. 2014;3(4):324-32. Epub 2014/11/11. doi: 10.1007/s13668-014-0104-4. PubMed PMID: 25383254; PubMed Central PMCID: PMCPMC4218969.
33. Derose KP, Fulcar MA, Acevedo R, Armenta G, Jiménez-Paulino G, Bernard CL, et al. An integrated urban gardens and peer nutritional counseling intervention to address food insecurity among people with HIV in the Dominican Republic. AIDS Education and Prevention. 2021;33(3):187-201. doi: 10.1521/aeap.2021.33.3.187.
34. Devers KJ, Frankel RM. Study design in qualitative research--2: Sampling and data collection strategies. Education for Health 2000;13(2):263-71. Epub 2004/01/27. doi: 10.1080/13576280050074543. PubMed PMID: 14742088.

35. Braun V, Clarke V. Using thematic analysis in psychology. Qualitative Research in Psychology. 2006;3(2):77-101. doi: 10.1191/1478088706qp063oa. PubMed PMID: 223135521.
36. Dedoose. Web application for managing, analyzing, and presenting qualitative and mixed method research data. Version 8.0.35 ed. Los Angeles, CA: SocioCultural Research Consultants, LLC; 2018.
37. Leventhal S. A gap between ideals and reality: The right to health and the inaccessibility of healthcare for Haitian migrant workers in the Dominican Republic. Emory International Law Review. 2013;27(2).
38. Keys HM, Keys HM, Kaiser BN, Foster JW, Burgos Minaya RY. Perceived discrimination, humiliation, and mental health: A mixed-methods study among Haitian migrants in the Dominican Republic. Ethnicity & Health. 2015;20(3):219-40. doi: 10.1080/13557858.2014.907389.
39. PAHO. Indicadores básicos 2019: Tendencias de la salud en las Américas. Washington, DC: Pan American Health Organization, 2019 October 1, 2019. Report No.
40. Ministerio de Salud Pública (MSP). Plan nacional de prevencion y control de las enfermedades no transmisibles 2019-2024. Santo Domingo, Republic Dominicana Ministerio de Salud Pública y Asistencia Social, 2019.
41. Ministerio de Salud Pública (MSP). Republic Dominicana - Plan estrategico control integrado ECNT Santo Domingo, Republica Dominicana2014 [cited 2020 October 21]. Available from: https://www.iccp-portal.org/republica-dominicana-plan-estrategico-control-integrado-ecnt.
42. Seguro National de Salud. SeNaSA, el SNS, JCE, y CONAVIHSIDA firman acuerdo interinstitucional para afiliar al régimen subsidiado personas que viven con VIH Santo Domingo, Republica Dominicana: Seguro Nacional de Salud (SENASA); 2019 [cited 2021 October 21]. Available from:

https://www.arssenasa.gob.do/index.php/blog/2019/10/15/senasa-el-sns-jce-y-conavihsida-firman-acuerdo-interinstitucional-para-afiliar-al-regimen-subsidiado-personas-que-viven-con-vih/.

43. Barrington C, Kerrigan D, Ureña FIC, Brudney K. La vida normal: living with HIV in Santo Domingo, Dominican Republic. Culture, Health & Sexuality. 2018;20(1):40-54. doi: 10.1080/13691058.2017.1323350.

44. Wallace DD, Gonzalez Rodriguez H, Walker E, Dethlefs H, Dowd RA, Filipi L, et al. Types and sources of social support among adults living with type 2 diabetes in rural communities in the Dominican Republic. Global Public Health. 2019;14(1):135-46. doi: 10.1080/17441692.2018.1444782.

Dear Editorial team,

We thank you for the opportunity to resubmit the manuscript: “The co-management of HIV and chronic non-communicable diseases in the Dominican Republic: A qualitative study” for exclusive consideration in *PLOS One*.

Our response to reviewers is found below.

Editorial Notes

When submitting your revision, we need you to address these additional requirements.

1. *Please ensure that your manuscript meets PLOS ONE's style requirements, including those for file naming. The PLOS ONE style templates can be found at https://journals.plos.org/plosone/s/file?id=wjVg/PLOSOne_formatting_sample_main_body.pdf and https://journals.plos.org/plosone/s/file?id=ba62/PLOSOne_formatting_sample_title_authors_affiliations.pdf*

    - We have ensured that we followed the PLOS ONE style requirements.

2. *Please include a complete copy of PLOS' questionnaire on inclusivity in global research in your revised manuscript. Our policy for research in this area aims to improve transparency in the reporting of research performed outside of researchers' own country or community. The policy applies to researchers who have travelled to a different country to conduct research, research with Indigenous populations or their lands, and research on cultural artefacts. The questionnaire can also be requested at the journal's discretion for any other submissions, even if these conditions are not met. Please find more information on the policy and a link to download a blank copy of the questionnaire here: https://journals.plos.org/plosone/s/best-practices-in-research-reporting. Please upload a completed version of your questionnaire as Supporting Information when you resubmit your manuscript.*
    - The questionnaire on inclusivity in global research was completed and uploaded.

3. *Please provide additional details regarding participant consent. In the ethics statement in the Methods and online submission information, please ensure that you have specified whether: 1) whether the ethics committee approved the verbal/oral consent procedure, 2) why written consent could not be obtained, and 3) how verbal/oral consent was recorded. If your study included minors, please state whether you obtained consent from parents or guardians in these cases. If the need for consent was waived by the ethics committee, please include this information.*
    - Consent information was updated. We specifically added text on why we conducted verbal consent (for literacy and accessibility purposes).

4. *In your Data Availability statement, you have not specified where the minimal data set underlying the results described in your manuscript can be found. PLOS defines a study's minimal data set as the underlying data used to reach the conclusions drawn in the manuscript and any additional data required to replicate the reported study findings in their entirety. All PLOS journals require that the minimal data set be made fully available. For more information about our data policy, please see http://journals.plos.org/plosone/s/data-availability.*

   "Upon re-submitting your revised manuscript, please upload your study's minimal underlying data set as either Supporting Information files or to a stable, public repository and include the relevant URLs, DOIs, or accession numbers within your revised cover letter. For a list of acceptable repositories, please see http://journals.plos.org/plosone/s/data-availability#loc-recommended-repositories. Any potentially identifying patient information must be fully anonymized.

   Important: If there are ethical or legal restrictions to sharing your data publicly, please explain these restrictions in detail. Please see our guidelines for more information on what we consider unacceptable restrictions to publicly sharing data: http://journals.plos.org/plosone/s/data-availability#loc-unacceptable-data-access-restrictions. Note that it is not acceptable for the authors to be the sole named individuals responsible for ensuring data access.

   We will update your Data Availability statement to reflect the information you provide in your cover letter.

Additional comment from Editorial team November 16, 2022:

1. We note your updated Data Availability statement as follows:
"Data underlying the results cannot be shared publicly to protect participant privacy and confidentiality. Because we did not state in the consent form that the data could be made available to outside researchers, our IRB has informed us that we cannot make the data available outside the study team."

In this instance it seems there may be acceptable restrictions in place that prevent the public sharing of your minimal data, however, in line with our goal of ensuring long-term data availability to all interested researchers, PLOS' Data Policy states that authors cannot be the sole named individuals responsible for ensuring data access (http://journals.plos.org/plosone/s/data-availability#loc-acceptable-data-sharing-methods).

Data requests to a non-author institutional point of contact, such as a data access or ethics committee, helps guarantee long term stability and availability of data. Providing interested researchers with a durable point of contact ensures data will be accessible even if an author changes email addresses, institutions, or becomes unavailable to answer requests.

Before we proceed with your manuscript, please address the following prompts:

a) If there are ethical or legal restrictions on sharing a de-identified data set, please explain the restrictions in detail (e.g., data contain potentially identifying or sensitive patient information) and who has imposed them (e.g., a Research Ethics Committee or Institutional Review Board, etc.).

Please also provide non-author contact information (phone/email/hyperlink) for a data access committee, ethics committee, or other institutional body to which data requests may be sent. If applicable, please also provide any necessary information which interested researchers would need when requesting access to data in order to obtain the minimal data set for your study.

We will update your Data Availability statement on your behalf to reflect the information you provide.

- We have added a statement on data availability of the following: "The data upon which this manuscript is based are in-depth interview transcripts from a small sample of marginalized and stigmatized people with HIV and contain highly sensitive data that are identifiable via inference. Thus, sharing them would violate the promise of confidentiality made to participants during informed consent. Because we did not state in the consent form that the data could be made available to outside researchers, the RAND Human Subjects Protection Committee (HSPC) has informed us that we cannot make the data available outside the study team. Queries regarding data access can be addressed by the RAND HSPC by contacting Rebecca Collins, HSPC Chair (collins@rand.org)"
- Therefore, we cannot provide a URL for public access to the data.

Comments to the Author

Reviewer #1:

The co-management of HIV and chronic non-communicable diseases in the Dominican Republic: A qualitative study

Review:

Manuscript presented an important topic and problem of high significance. The authors intended to explore PLWH lived experiences in co-managing multiple chronic conditions. There are significant and minor revisions suggested and described in each section.

Background
There is the mention of food insecurity in the background but not much is discussed about it. Is this a problem in Dominican Republic? Who is severely affected by food insecurity? Context can help guide the reason for including food insecurity.

- Thank you for this critique. We have added additional context and information about food security in lines 74-81. We included additional citations that focus on the role food insecurity has on the management of different conditions (HIV, NCDs).

Line 25-27 - "Of the individuals living with HIV in the Dominican Republic, 82% know their status and 51% of people living HIV, regardless of status, are on antiretroviral therapy (ART)"
This sentence is not clear. "Regardless of status" Does this mean that there are some without

HIV and are accounted for in the 51%? Please assist the reader to understand the reason why it is important to include those without HIV and ART.

- The line in question was edited for clarity.

Line 31-38 described overall trends of NCDs with little mention of the current situation in Dominican Republic. For example, are the age groups of those with HIV changing to an older population? This can help motivate the significance of the research area.

- Lines 34-37 are specifically referring to the overall trends in the Dominican Republic and lines 21-29 refers to the current situation in the Dominican Republic in regard to HIV.

Line 45-46: "At the individual level, those with multiple conditions require greater healthcare utilization and therefore incur greater healthcare costs than individuals with one or zero chronic conditions."
Is this to be required or studies have found association between number of chronic conditions and higher utilization and cost?

- We have added a citation to validate the argument.

Lines 39-69: Appreciated the background information but this section was hard to follow and also was too general. It would be helpful to narrow the focus to HIV and NCD. Discussing the conditions separately was very hard to tie back to the objective of the study. Please review this section.

- Thank you for your comments. We reviewed this section and note that it is focused on the discussion of HIV and NCDs, specifically the need to consider the co-management of these conditions. Lines 17-37 do present the context of these conditions separately; however, the section indicates that the critique is focused on the interplay of these conditions. Therefore, we did not make any edits beyond those for clarity and readability.

Line 79 mentions the use of the socio ecological model to inform the study. In the background section, one expects to learn how the model has been used in other studies and then its choice is defended in the methods section. Was the model used to develop the interview guide? Why was this the model of choice?

- The socioecological model is a framing for the data collection strategy and analysis. It's been used extensively in public health, but to clarify for the reader on how we used it, we added a sentence to the Data Collection subsection that reads as follows: "Using a socioecological model we framed question to understand individual, interpersonal, organizational, and political barriers and facilitators to managing multiple chronic conditions." Also, in lines 66-68 we reference the need to account for "individual, interpersonal, and structural facilitators and barriers to disease co-management…" which alludes to the socioecological model.

Methods
Line 107- there is a mention of a "ProMeSA study" this is the first time it is mentioned and the last time. What was it? How was it related to the intervention?

- The ProMeSA study is the intervention study. We added language to clarify that the intervention study is named ProMeSA. Related information about the study was masked for review, however, we have cited the intervention for more information.
- The endline interview are the data used for the present substudy, as explained in lines 109-117.

Why did the researchers decide to interview participants in the intervention group? Since they were interested in barriers and facilitators for co-management

of NCDs, what was specific about the intervention group?

- All participants were part of an intervention, thus there is not a differentiation between a non-intervention and intervention group. The intervention, ProMeSA, components was described in lines 95-101. We chose to do a qualitative evaluation of the intervention overall, but also asked about barriers and facilitators for co-managing multiple chronic conditions. Therefore, as the main study only targeted HIV-specific outcomes, we could ask about NCD self-management in the qualitative sub-study

Recommend use the COREQ (COnsolidated criteria for REporting Qualitative research) Checklist as a guide in what to be reported.

- Thank you for the recommendation, we have filled out the COREQ checklist.

Results
Line 117: mentions of recruitment until saturation was reached. After how many participants was saturation reached?

- At approximately 20 participants we did reach thematic saturation, therefore we stopped recruitment at 21 participants.

Generally: although food insecurity is mentioned in the background, nothing seemed to have been explored in the topic.

- Thank you for this comment. The intervention study enrolled only participants who had moderate or severe food insecurity (per the Latin American Food Insecurity Scale). As this is the case, all narratives (i.e., data) are from this lens. We have added text in the results to this effect (lines 179-181). We also ensured to emphasize this throughout the manuscript to strengthen this foundation.

Since the study was focused on co-management of HIV and chronic NCDs, it is important that the results mostly focus on this aspect. The sections beginning line 174 -239 seem to be out of the topic of discussion.

- Thank you for this comment. We understand that the focus is on the co-management of HIV and NCDs. We felt that it was important to provide some context for what it is like to

live with HIV for adults who are food insecure in general, and equally what it is like to live with NCDs as an adult experiencing food insecurity.

Minor: In table 2, authors should be consistent with abbreviations (T2D was used in some places and then one would see "type 2 diabetes" later)

- Thank you. We have corrected Table 2 for consistency.

Line 293 -294 "The lack of education was also tied to how participants experienced their condition as being "sensitive" and "unpredictable" from one day to the next."
This sentence is not clear. Consider revising.

- Thank you. We clarified what we meant by lack of education. The sentence now reads as follows: The lack of education on <u>NCD self-management</u> was also tied to how participants experienced their condition as being "sensitive" and "unpredictable" from one day to the next.

Line 327-337: this section was confusing. It is not clear what was said by participants.

- Thank you for your note. This first paragraph under structural level concerns focused on summarizing results from demographic variables (see Table 1). We also made a note throughout this paragraph that compared how participants spoke about their experiences with accessing the health care system. These experiences did not change much between those who were insured or not, particularly when managing NCDs. Therefore, we did not pull specific quotes. Rather we highlighted exemplar experiences for the remaining parts of the subsection.

Line 332-334 is also confusing. What information should the reader get from this sentence? "Of the five participants who were without health insurance …….three were the participants of Haitian descent"
Why was it important for the authors to add this sentence? Should the reader be introduced to the aspect earlier in the background?

- At the beginning of the results section and Table 1, we have an ancestry break down in which three participants from our total pool of participants were of Haitian descent. We brought up this sentence of lack of health insurance in the results and further link to potential implications of this in second paragraph of the discussion section. In the discussion we note potential barriers for Haitians in the Dominican Republic to gain access to health insurance. We did not bring this up in the background because this was not an a priori assumption and it fit best to link the results to the discussion.

Discussion
The discussion section provided information about that healthcare system that should have been provided in the background section. Consider revising this section to discuss the results and next steps.

- Thank you for this comment. The reason we have included the background information on the healthcare system in the discussion rather than in the background is because it was related to how participants described their experiences. This was not an a priori assumption, and we wanted to recognize this as linked to the data.

Reviewer #2:

1. *Well done overall. The presentation and discussion on the key facilitators and barriers for individuals living with HIV and co-occurring NCDs was informative - especially Table 2.*

    - Thank you for the comments. We appreciate that the manuscript was considered relevant and informative.

2. *Please re-read the manuscript as there are a few grammatical errors within the text.*

    - We have re-read the manuscript and addressed grammatical errors.
