# Supplementary material for: The co-management of HIV and chronic non-communicable diseases in the Dominican Republic: A qualitative study
Source: PLoS One. 2023 Jul 13;18(7):e0288583. doi: 10.1371/journal.pone.0288583 (PMC10343047; doi:10.1371/journal.pone.0288583)
Supplement: Attachment — Submitted filename: Response-to-reviewers.docx [file pone.0288583.s003.docx]

Dear Editorial team,

We thank you for the opportunity to resubmit the manuscript: “The co-management of HIV and chronic non-communicable diseases in the Dominican Republic: A qualitative study” for exclusive consideration in *PLOS One*.

Our response to reviewers is found below.

**Editorial Notes**

*When submitting your revision, we need you to address these additional requirements.*

1. *Please ensure that your manuscript meets PLOS ONE's style requirements, including those for file naming. The PLOS ONE style templates can be found at*
   [*https://journals.plos.org/plosone/s/file?id=wjVg/PLOSOne_formatting_sample_main_body.pdf*](https://journals.plos.org/plosone/s/file?id=wjVg/PLOSOne_formatting_sample_main_body.pdf) *and* 
   [*https://journals.plos.org/plosone/s/file?id=ba62/PLOSOne_formatting_sample_title_authors_affiliations.pdf*](https://journals.plos.org/plosone/s/file?id=ba62/PLOSOne_formatting_sample_title_authors_affiliations.pdf)

- We have ensured that we followed the PLOS ONE style requirements.

2. *Please include a complete copy of PLOS’ questionnaire on inclusivity in global research in your revised manuscript. Our policy for research in this area aims to improve transparency in the reporting of research performed outside of researchers’ own country or community. The policy applies to researchers who have travelled to a different country to conduct research, research with Indigenous populations or their lands, and research on cultural artefacts. The questionnaire can also be requested at the journal’s discretion for any other submissions, even if these conditions are not met.  Please find more information on the policy and a link to download a blank copy of the questionnaire here: https://journals.plos.org/plosone/s/best-practices-in-research-reporting. Please upload a completed version of your questionnaire as Supporting Information when you resubmit your manuscript.*
   - The questionnaire on inclusivity in global research was completed and uploaded.
3. *Please provide additional details regarding participant consent. In the ethics statement in the Methods and online submission information, please ensure that you have specified whether: 1) whether the ethics committee approved the verbal/oral consent procedure, 2) why written consent could not be obtained, and 3) how verbal/oral consent was recorded. If your study included minors, please state whether you obtained consent from parents or guardians in these cases. If the need for consent was waived by the ethics committee, please include this information.*
   - Consent information was updated. We specifically added text on why we conducted verbal consent (for literacy and accessibility purposes).
4. *In your Data Availability statement, you have not specified where the minimal data set underlying the results described in your manuscript can be found. PLOS defines a study's minimal data set as the underlying data used to reach the conclusions drawn in the manuscript and any additional data required to replicate the reported study findings in their entirety. All PLOS journals require that the minimal data set be made fully available. For more information about our data policy, please see* [*http://journals.plos.org/plosone/s/data-availability*](http://journals.plos.org/plosone/s/data-availability)*.*

*"Upon re-submitting your revised manuscript, please upload your study’s minimal underlying data set as either Supporting Information files or to a stable, public repository and include the relevant URLs, DOIs, or accession numbers within your revised cover letter. For a list of acceptable repositories, please see http://journals.plos.org/plosone/s/data-availability#loc-recommended-repositories. Any potentially identifying patient information must be fully anonymized.*

*Important: If there are ethical or legal restrictions to sharing your data publicly, please explain these restrictions in detail. Please see our guidelines for more information on what we consider unacceptable restrictions to publicly sharing data: http://journals.plos.org/plosone/s/data-availability#loc-unacceptable-data-access-restrictions. Note that it is not acceptable for the authors to be the sole named individuals responsible for ensuring data access.*

*We will update your Data Availability statement to reflect the information you provide in your cover letter.*

***Additional comment from Editorial team November 16, 2022:***

*1. We note your updated Data Availability statement as follows:
"Data underlying the results cannot be shared publicly to protect participant privacy and confidentiality. Because we did not state in the consent form that the data could be made available to outside researchers, our IRB has informed us that we cannot make the data available outside the study team."

In this instance it seems there may be acceptable restrictions in place that prevent the public sharing of your minimal data, however, in line with our goal of ensuring long-term data availability to all interested researchers, PLOS’ Data Policy states that authors cannot be the sole named individuals responsible for ensuring data access (*[*http://journals.plos.org/plosone/s/data-availability#loc-acceptable-data-sharing-methods*](http://journals.plos.org/plosone/s/data-availability#loc-acceptable-data-sharing-methods)*).

Data requests to a non-author institutional point of contact, such as a data access or ethics committee, helps guarantee long term stability and availability of data. Providing interested researchers with a durable point of contact ensures data will be accessible even if an author changes email addresses, institutions, or becomes unavailable to answer requests.

Before we proceed with your manuscript, please address the following prompts:

a) If there are ethical or legal restrictions on sharing a de-identified data set, please explain the restrictions in detail (e.g., data contain potentially identifying or sensitive patient information) and who has imposed them (e.g., a Research Ethics Committee or Institutional Review Board, etc.). Please also provide non-author contact information (phone/email/hyperlink) for a data access committee, ethics committee, or other institutional body to which data requests may be sent. If applicable, please also provide any necessary information which interested researchers would need when requesting access to data in order to obtain the minimal data set for your study.

We will update your Data Availability statement on your behalf to reflect the information you provide.*

- We have added a statement on data availability of the following: “The data upon which this manuscript is based are in-depth interview transcripts from a small sample of marginalized and stigmatized people with HIV and contain highly sensitive data that are identifiable via inference. Thus, sharing them would violate the promise of confidentiality made to participants during informed consent. Because we did not state in the consent form that the data could be made available to outside researchers, the RAND Human Subjects Protection Committee (HSPC) has informed us that we cannot make the data available outside the study team. Queries regarding data access can be addressed by the RAND HSPC by contacting Rebecca Collins, HSPC Chair ([collins@rand.org](mailto:collins@rand.org))”
- Therefore, we cannot provide a URL for public access to the data.

**Comments to the Author**

**Reviewer #1:**

*The co-management of HIV and chronic non-communicable diseases in the Dominican
Republic: A qualitative study

Review:

Manuscript presented an important topic and problem of high significance. The authors intended to explore PLWH lived experiences in co-managing multiple chronic conditions. There are significant and minor revisions suggested and described in each section.

Background
There is the mention of food insecurity in the background but not much is discussed about it. Is this a problem in Dominican Republic? Who is severely affected by food insecurity? Context can help guide the reason for including food insecurity.*

- Thank you for this critique. We have added additional context and information about food security in lines 74-81. We included additional citations that focus on the role food insecurity has on the management of different conditions (HIV, NCDs).

*Line 25-27 - “Of the individuals living with HIV in the Dominican Republic, 82% know their status and 51% of people living HIV, regardless of status, are on antiretroviral therapy (ART)”
This sentence is not clear. “Regardless of status” Does this mean that there are some without HIV and are accounted for in the 51%? Please assist the reader to understand the reason why it is important to include those without HIV and ART.*

- The line in question was edited for clarity.

*Line 31-38 described overall trends of NCDs with little mention of the current situation in Dominican Republic. For example, are the age groups of those with HIV changing to an older population? This can help motivate the significance of the research area.*

- Lines 34-37 are specifically referring to the overall trends in the Dominican Republic and lines 21-29 refers to the current situation in the Dominican Republic in regard to HIV.

*Line 45-46: “At the individual level, those with multiple conditions require greater healthcare utilization and therefore incur greater healthcare costs than individuals with one or zero chronic conditions.”
Is this to be required or studies have found association between number of chronic conditions and higher utilization and cost?*

- We have added a citation to validate the argument.

*Lines 39-69: Appreciated the background information but this section was hard to follow and also was too general. It would be helpful to narrow the focus to HIV and NCD. Discussing the conditions separately was very hard to tie back to the objective of the study. Please review this section.*

- Thank you for your comments. We reviewed this section and note that it is focused on the discussion of HIV and NCDs, specifically the need to consider the co-management of these conditions. Lines 17-37 do present the context of these conditions separately; however, the section indicates that the critique is focused on the interplay of these conditions. Therefore, we did not make any edits beyond those for clarity and readability.

*Line 79 mentions the use of the socio ecological model to inform the study. In the background section, one expects to learn how the model has been used in other studies and then its choice is defended in the methods section. Was the model used to develop the interview guide? Why was this the model of choice?*

- The socioecological model is a framing for the data collection strategy and analysis. It’s been used extensively in public health, but to clarify for the reader on how we used it, we added a sentence to the Data Collection subsection that reads as follows: “Using a socioecological model we framed question to understand individual, interpersonal, organizational, and political barriers and facilitators to managing multiple chronic conditions.” Also, in lines 66-68 we reference the need to account for “individual, interpersonal, and structural facilitators and barriers to disease co-management…” which alludes to the socioecological model.

*Methods
Line 107- there is a mention of a “ProMeSA study” this is the first time it is mentioned and the last time. What was it? How was it related to the intervention?*

- The ProMeSA study is the intervention study. We added language to clarify that the intervention study is named ProMeSA. Related information about the study was masked for review, however, we have cited the intervention for more information.
- The endline interview are the data used for the present substudy, as explained in lines 109-117.

*Why did the researchers decide to interview participants in the intervention group? Since they were interested in barriers and facilitators for co-management*

*of NCDs, what was specific about the intervention group?*

- All participants were part of an intervention, thus there is not a differentiation between a non-intervention and intervention group. The intervention, ProMeSA, components was described in lines 95-101. We chose to do a qualitative evaluation of the intervention overall, but also asked about barriers and facilitators for co-managing multiple chronic conditions. Therefore, as the main study only targeted HIV-specific outcomes, we could ask about NCD self-management in the qualitative sub-study

*Recommend use the COREQ (COnsolidated criteria for REporting Qualitative research) Checklist as a guide in what to be reported.*

- Thank you for the recommendation, we have filled out the COREQ checklist.

*Results
Line 117: mentions of recruitment until saturation was reached. After how many participants was saturation reached?*

- At approximately 20 participants we did reach thematic saturation, therefore we stopped recruitment at 21 participants.

*Generally: although food insecurity is mentioned in the background, nothing seemed to have been explored in the topic.*

- Thank you for this comment. The intervention study enrolled only participants who had moderate or severe food insecurity (per the Latin American Food Insecurity Scale). As this is the case, all narratives (i.e., data) are from this lens. We have added text in the results to this effect (lines 179-181). We also ensured to emphasize this throughout the manuscript to strengthen this foundation.

*Since the study was focused on co-management of HIV and chronic NCDs, it is important that the results mostly focus on this aspect. The sections beginning line 174 -239 seem to be out of the topic of discussion.*

- Thank you for this comment. We understand that the focus is on the co-management of HIV and NCDs. We felt that it was important to provide some context for what it is like to live with HIV for adults who are food insecure in general, and equally what it is like to live with NCDs as an adult experiencing food insecurity.

*Minor: In table 2, authors should be consistent with abbreviations (T2D was used in some places and then one would see “type 2 diabetes” later)*

- Thank you. We have corrected Table 2 for consistency.

*Line 293 -294 “The lack of education was also tied to how participants experienced their condition as being “sensitive” and “unpredictable” from one day to the next.”
This sentence is not clear. Consider revising.*

- Thank you. We clarified what we meant by lack of education. The sentence now reads as follows: The lack of education on NCD self-management was also tied to how participants experienced their condition as being “sensitive” and “unpredictable” from one day to the next.

*Line 327-337: this section was confusing. It is not clear what was said by participants.*

- Thank you for your note. This first paragraph under structural level concerns focused on summarizing results from demographic variables (see Table 1). We also made a note throughout this paragraph that compared how participants spoke about their experiences with accessing the health care system. These experiences did not change much between those who were insured or not, particularly when managing NCDs. Therefore, we did not pull specific quotes. Rather we highlighted exemplar experiences for the remaining parts of the subsection.

*Line 332-334 is also confusing. What information should the reader get from this sentence? “Of the five participants who were without health insurance …….three were the participants of Haitian descent”
Why was it important for the authors to add this sentence? Should the reader be introduced to the aspect earlier in the background?*

- At the beginning of the results section and Table 1, we have an ancestry break down in which three participants from our total pool of participants were of Haitian descent. We brought up this sentence of lack of health insurance in the results and further link to potential implications of this in second paragraph of the discussion section. In the discussion we note potential barriers for Haitians in the Dominican Republic to gain access to health insurance. We did not bring this up in the background because this was not an a priori assumption and it fit best to link the results to the discussion.

*Discussion
The discussion section provided information about that healthcare system that should have been provided in the background section. Consider revising this section to discuss the results and next steps.*

- Thank you for this comment. The reason we have included the background information on the healthcare system in the discussion rather than in the background is because it was related to how participants described their experiences. This was not an a priori assumption, and we wanted to recognize this as linked to the data.

**Reviewer #2:**

1. *Well done overall. The presentation and discussion on the key facilitators and barriers for individuals living with HIV and co-occurring NCDs was informative - especially Table 2.*

- Thank you for the comments. We appreciate that the manuscript was considered relevant and informative.

2. *Please re-read the manuscript as there are a few grammatical errors within the text.*

- We have re-read the manuscript and addressed grammatical errors.
