# Supplementary material for: The co-management of HIV and chronic non-communicable diseases in the Dominican Republic: A qualitative study
Source: PLoS One. 2023 Jul 13;18(7):e0288583. doi: 10.1371/journal.pone.0288583 (PMC10343047; doi:10.1371/journal.pone.0288583)
Supplement: Attachment — Submitted filename: Response-to-reviewers.docx [file pone.0288583.s005.docx]

June 20, 2023

Dear Editorial Team and Reviewers,

We thank you for the opportunity to resubmit the manuscript: “The co-management of HIV and chronic non-communicable diseases in the Dominican Republic: A qualitative study” for exclusive consideration in *PLOS One*.

Our response to reviewers is found below.

**Comments to the Author**

**Reviewer #3:**

*Thank you for a great manuscript providing key insights into a growing health challenge of high significance that is often understudied. With health systems grappling with an increasing double burden of communicable and non-communicable disease, co and multi morbid health conditions, the findings of the study provide key insights to informing health policy and planning within the context of competing resources. The paper is keenly needed but in its present form, i am sorry to say it leaves the reader confused. My comments and suggestions are attached in the manuscript. I hope this helps.

Reply:*

- We thank the reviewer for their suggestions. We have addressed several comments and edited the manuscript to improve clarity, where needed.
- Edits on Abstract:
  - Food insecurity is an inclusion criterion as stated; therefore, the sentence was not edited. We kept the clause “we explored participant lived experiences co-managing…” because the focus of this manuscript is on the co-management piece, which is different from the inclusion criteria sentence.
  - Made small revisions to improve clarity and parallel structure to the abstract.
- Edits on Introduction:
  - The “Introduction” is a header, not a title; therefore, it stayed as is to ensure we are in line with PLOS One’s template.
  - We understand that the reviewer believes that the introduction is long, however, we are providing the necessary context for the study. We preferred to keep the flow as is to reflect the flow of the results section, starting with HIV, moving into NCDs, and then gaps in information in co-management.
  - We addressed general edits to improve clarity.
- Edits on Materials and Methods
  - Thank you. We addressed grammatical errors and added some phrasing to improve clarity.
  - There were questions on some phrasing – RAND Corporation is the name of the company – RAND is not an acronym therefore we kept it as the official name of the organization. Semi-structured, in-depth interviews is the description of the methods. For example, in-depth interviews means one-on-one interviews.
  - Prior reviewers requested example questions and those were provided. Interview guide is in Spanish (not translated); however, we have attached the English version of the interview guide.
  - Data collection processes were described according to COREQ guidelines.
  - We placed the number of interviews (21) in the results.
- Edits on Results
  - None.
- Edits on Discussion
  - Made line edits to improve clarity throughout the document. Rearranged the discussion to focus on themes in a similar order to the results section to improve clarity.

**Reviewer #4:**

*Setting & participants: Were there any refusals? Any patients invited to participate who refused to consent?*

*Any bias: Was there a possibility of undue influence to participate if patients were recruited where they were getting care and the interventions?.*

- Thank you for the comments. Yes, participants were able to refuse to participate in the study. Through the consent process, patients also got assurances that refusal to participate would not affect their care. Specific language in the consent is: “Your participation in this interview is completely voluntary. You do not have to participate if you do not want to and you can stop the interview at any time or withdraw your consent to be in the study for any reason without penalty or repercussions.”

*2. Identification & recruitment: How exactly were the 21 patients:*

*- Identified e.g. through hospital datasets? Describe this step by step*

*-Invited to participate? E.g. via telephone calls? During their regular clinic visits? And so on*

- Thank you for your questions. Participants were identified through the sampling frame from the intervention and based on clinic records. We provide details of this under settings and participants: “For the qualitative endline sub-study, we used in-person purposive sampling to select intervention participants who also had a chronic NCD (i.e., hypertension, diabetes, prediabetes, high cholesterol, obesity) according to previously collected data. We purposively recruited approximately equal numbers of men and women.”

*3. Verbal consent: The participants were engaged in-person. What does this mean ‘verbal consent to account for literacy and accessibility’?*

*How was the verbal consent carried out? Describe it step by step.*

*How was it captured by the research team?*

- Thank you. Verbal consent rather than written consent was done to account for variability in literacy (i.e., not everyone can read). To be explicit about that, we are leaving the phrasing as is.
- Verbal consent was carried out in person, the consent form read aloud, and at the end the participant could verbally indicate their consent to participate.

*4. 'Example questions included: how are you managing your HIV? How are you managing your chronic condition (T2D, high blood pressure)? Which of your health conditions worries you the most?' Provide the study tool as an appendage?*

- We do include sample questions, and have included an English version of the guide in the appendix.

*5. 'After each interview, interviewers wrote field notes about the interview setting, key themes of the interview, observations for the following interviews, and if any themes may have reached saturation.' Is it feasible to actually deduce data saturation at the end of each interview? How did the interviewers do it within short time spans? Add those details.*

- Field notes are always done immediately after each interview. We added the word “immediately” to clarify time lag.
- We simplified the language and removed themes reaching saturation to better reflect the ana
- We are unsure what the question is asking about how interviewer do within short time spans. Does this refer to the field notes or to how many interviews are done? The number of interviews done were based on the day and clinic and we indicate that the 21 interviews were conducted over the course of 4 months (October 2019-February 2020).

*6. Results. Very rich data reported. Thank you*

- Thank you, we appreciate that.

*7. Discussion points are appreciated, however, the flow can be improved. The ideas are there but the reader gets totally lost. Arrange the discussion to closely match the layout of the study findings
a.
-HIV management
-Chronic NCD management

b. Management of multiple conditions
-Individual
-Interpersonal
-Structural level*

- Thank you for your comments. The discussion section was revised as needed.

*8. Nice conclusion. Edit to capture that these were '...Perspective of PATIENTS…not just 'adults experiencing food insecurity...'*

- Edited in the strengths and conclusions section.

*9. Data availability. Can an interested researcher reach out to your corresponding author and/or their IRB if they have queries about your data and its analysis? Can you maintain possibility of future interactions with other researchers on data, associated tools, and data analysis?*

- Yes, inquiries can be addressed on a rolling basis. The research team is happy to talk through the analysis and discuss data structure. However, given that IRB and ethics approvals were needed across countries and different institutions, we cannot place the data in a repository to be widely shared, as it violates ethics agreements.

*10. Acknowledgment: 'The authors thank the participants for their generosity in providing their time as well as the health clinic staff and providers that participated.'
What was the role of health clinic staff and providers in this study? Were they involved in recruitment? Interviews? Any biases?*

- No, health clinic staff were not involved in the recruitment. We clarified this in the acknowledgements. The research staff were independent of the clinic. We thank the health staff for allowing the team space to conduct the research.
